# Anticancer Properties of Graviola (*Annona muricata*): A Comprehensive Mechanistic Review

**DOI:** 10.1155/2018/1826170

**Published:** 2018-07-30

**Authors:** Islam Rady, Melissa B. Bloch, Roxane-Cherille N. Chamcheu, Sergette Banang Mbeumi, Md Rafi Anwar, Hadir Mohamed, Abiola S. Babatunde, Jules-Roger Kuiate, Felicite K. Noubissi, Khalid A. El Sayed, G. Kerr Whitfield, Jean Christopher Chamcheu

**Affiliations:** ^1^Department of Dermatology, School of Medicine and Public Health, University of Wisconsin-Madison, WI 53706, USA; ^2^School of Pharmaceutical and Toxicological Sciences, College of Pharmacy, University of Louisiana at Monroe, Monroe, LA 71201, USA; ^3^Madison West High School, 30 Ash St, Madison, WI 53726, USA; ^4^Division for Research and Innovation, POHOFI Inc., P.O. Box 44067, Madison, WI 53744, USA; ^5^Department of Biochemistry, Faculty of Science, University of Mansoura, Mansoura, Egypt; ^6^Department of Hematology, University of Ilorin, Ilorin, Nigeria; ^7^Department of Biochemistry, Faculty of Sciences, University of Dschang, Dschang, Cameroon; ^8^Section for Research and Innovation, POHOFCAM, P.O. Box 175, Kumba, Cameroon; ^9^Department of Biology/RCMI, Jackson State University, 1400 J R Lynch, 429 JAP, Jackson, MS 39217, USA; ^10^Department of Basic Medical Sciences, University of Arizona College of Medicine-Phoenix, Phoenix, AZ 85004, USA

## Abstract

Graviola (*Annona muricata*) is a small deciduous tropical evergreen fruit tree, belonging to the Annonaceae family, and is widely grown and distributed in tropical and subtropical regions around the world. The aerial parts of graviola have several functions: the fruits have been widely used as food confectionaries, while several preparations, especially decoctions of the bark, fruits, leaves, pericarp, seeds, and roots, have been extensively used in traditional medicine to treat multiple ailments including cancers by local communities in tropical Africa and South America. The reported therapeutic benefits of graviola against various human tumors and disease agents in in vitro culture and preclinical animal model systems are typically tested for their ability to specifically target the disease, while exerting little or no effect on normal cell viability. Over 212 phytochemical ingredients have been reported in graviola extracts prepared from different plant parts. The specific bioactive constituents responsible for the major anticancer, antioxidant, anti-inflammatory, antimicrobial, and other health benefits of graviola include different classes of annonaceous acetogenins (metabolites and products of the polyketide pathway), alkaloids, flavonoids, sterols, and others. This review summarize*s* the current understanding of the anticancer effects of *A. muricata* and its constituents on diverse cancer types and disease states, as well as efficacy and safety concerns. It also includes discussion of our current understanding of possible mechanisms of action, with the hope of further stimulating the development of improved and affordable therapies for a variety of ailments.

## 1. Introduction

Cancer is the second leading cause of mortality worldwide. Over 10 million new patients are diagnosed with cancer annually with over 6 million associated deaths, representing roughly 12% of worldwide mortality [1]. The occurrence of new cancer cases is expected to grow by about 70% over the next two decades and estimated to reach over 15 million new cases diagnosed annually by the year 2020 [2]. This rapid increase is due to both an aging and growing population, along with carcinogens, infections, genetic mutations, hormones, immune conditions, and the adoption of behavioral and dietary risk factors, such as smoking, unhealthy diet, physical inactivity, and environmental pollutants [3]. The risk factors may act singly or in concert to cause mutation of normal cells [4]. Many of these mutations alter the expression or activity of key gene products, causing unregulated cell division leading to cancer. Currently, the main cancer treatment modalities are surgery, radiation-based therapy, chemotherapy, gene therapy, and/or hormonal therapy, either singly or in combination [1]. The most commonly used chemotherapy drugs are antimetabolites, DNA-interacting agents, antitubulin agents, hormones, and molecular targeting agents, all of which work to destroy cancerous cells or limit their proliferation [5]. However, most cytotoxic drugs act on both cancerous and healthy cells and therefore elicit side effects such as hair loss, bone marrow suppression, drug resistance, gastrointestinal lesions, neurologic dysfunction, and cardiac toxicity [5]. Consequently, development of new anticancer agents with higher efficacy, selectivity, and little or no side effects is an urgent goal.

Natural products, especially phytochemicals, have been used to help mankind sustain health since the dawn of medicine [4]. Phytotherapy (also called herbalism or herbal medicine) has provided remedies for ailments, including cancer, to the present day [6]. Dietary phytochemicals have many built-in advantages over synthetic compounds due to their proven safety, low cost, and oral bioavailability [7]. However, it is only recently that researchers have begun to elucidate the mode of action of plant-derived agents at the molecular, cellular, and tissue level [8–10]. Many natural products have now been extensively researched, and numerous compounds have exhibited anticancer and other beneficial actions in modern controlled studies. Most anticancerous natural products interfere with the initiation, development, and progression of cancer by modulating various mechanisms including cellular proliferation, differentiation, apoptosis, angiogenesis, and metastasis [11].

**Figure 1:**
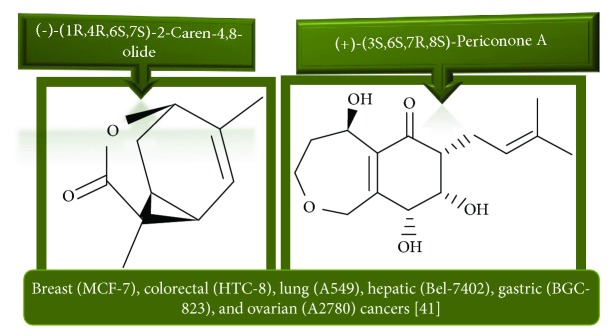
Chemical structure of compounds derived from fungal strain extracts and the cancers sensitive to them.

Extracts from *Annona muricata* (also known as graviola) are among a myriad of botanical products which have shown promising medicinal value [12–14]. Studies have linked *A. muricata-*derived compounds (Table 1 and Figure 1) to a variety of anticancer effects including cytotoxicity [15–18], induction of apoptosis [19–27], necrosis [28], and inhibition of proliferation [25, 29–31] on a variety of cancer cell lines, including breast [32], prostate [29], colorectal [25], lung [16], leukemia [33], renal [34], pancreatic [15], hepatic [24], oral [35], melanoma [36], cervical [37], and ovarian cancers [38]. Moreover, all aerial parts of this plant, including the bark, fruit, leaves, root, and seeds, are used as natural medicines in the tropics [39]. However, there is a need for more rigorous studies to establish safe and effective care regimes. This review summarizes the recent advances in the application and mechanisms of *A. muricata* extracts against several cancers both *in vitro* and *in vivo*.

## 2. Botanical Description and Distribution

**Figure 2:**
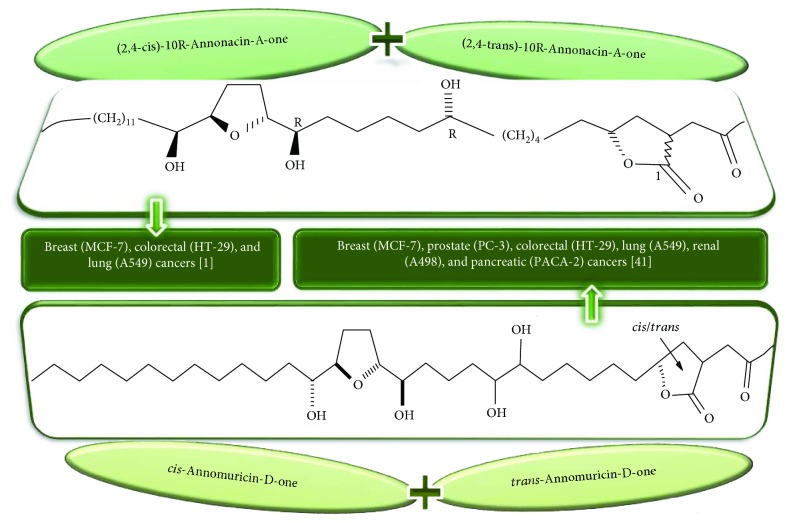
Chemical structures of two AGE combinations along with their targeted cancer phenotype.

*Annona muricata* is a lowland tropical, fruit-bearing tree of the family Annonaceae found in the rainforests of Africa, South America, and Southeast Asia. *A. muricata*, commonly known as soursop, graviola, guanabana, or Brazilian paw-paw, has large, glossy, dark green leaves [4, 40], with edible, green heart-shaped fruits [4, 41]. Soft, curved spines cover the leathery skin of the fruits, each of which may contain 55–170 black seeds distributed in a creamy white flesh with a characteristic aroma and flavor [41, 42]. All portions (leaves [16, 18, 31, 38, 43, 44], pericarp [24, 45, 46], fruits [4, 30, 47], seeds [47–50], and roots [27]) of *A. muricata* have been used in traditional medicine, but the most widely used in the preparations of traditional medical decoctions are stem barks, roots, seeds, and leaves [51, 52]. Coria-Téllez et al. have reported 212 bioactive compounds in *A. muricata* extracts [41]. Reports in the literature indicate that seventy-four of these bioactive compounds exhibit a variety of anticancer effects in preclinical cell culture and animal model systems. Several dozen annonaceous acetogenins have been studied (59 of which are listed alphabetically in Table 2, with key structural features summarized in Figure 2). Moreover, at least ten solvent extracts (Table 1) in addition to an extract from fungi (*Periconia* sp.) collected on *A. muricata* that contains bioactive compounds (Figure 1) have been tested for their anticancer properties and other health benefits.

*A. muricata*-derived preparations have been utilized to treat numerous ailments, making this plant an ethnomedically important species. In developing tropical countries including Africa, different parts of *A. muricata* are being used to treat conditions such as diabetes [53, 54], coughs, skin diseases [55], and cancers [25–27, 56–58]. Furthermore, in both Jamaica [59] and Trinidad [60], *A. muricata* is the most prevalently used herbal remedy in the treatment of most cancers. For example, in Jamaica, a large proportion of cancer patients use medicinal plants in self-medicating practices, with *A. muricata* being commonly used (along with *Petiveria alliacea*) for treating breast and prostate cancers, respectively [59].

*A. muricata* has also been used, mainly in developing tropical countries, for the treatment of arthritis [61], hypertension [62], snake bite [63], diarrhea [59], headache [64], and malaria [65]. In addition, it has been mentioned as an antimicrobial [66], antidiabetic [54], anti-inflammatory [67], antiprotozoan [68], antioxidant, insecticide [69], larvicide [70], and anticancer [71]. Although these uses of *A. muricata* strongly imply the presence of bioactive compounds with medical benefits, a full insight into the potential of *A. muricata* in the treatment of disease will require the identification of specific bioactive compounds and a scientifically rigorous demonstration of their ability to improve health outcomes.

## 3. Anticancer Effects

**Figure 3:**
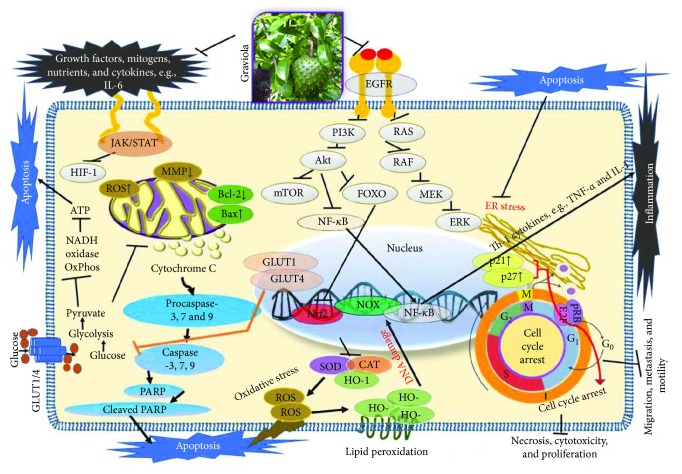
Overview of the molecular actions of *A. muricata* (graviola) leading to anticancer and other health benefits. Extracts of the different aerial parts of *A. muricata* using several solvents have been shown to induce cytotoxicity, cell cycle arrest, apoptosis, and necrosis and, conversely, to inhibit cancer cell motility, migration, metastasis, and proliferation. Other reported health benefits include antioxidant, anti-inflammatory, and immunomodulatory activities. Our current understanding is that graviola components modulate several cellular processes including inhibition of signaling pathways downstream of the epidermal growth factor receptor (EGFR), with others causing downregulation of phosphatidylinositol-4,5-bisphosphate 3-kinase (PI3K/Akt), RAS, NF-*κ*B, and JAK/STAT [31]. Further actions include inhibition of HIF-1*α*, GLUT1, and GLUT4 [28]; proinflammatory cytokine expression (inflammation); and generation of reactive oxygen species (ROS) via upregulatoin of enzyme systems like catalase (CAT), superoxide dismutase (SOD), and heme-oxygenase (HO-1) expression [39, 54, 89, 124].

More than 47% of current anticancer drugs on the market are natural products, their derivatives or natural product synthetic mimics, and more than 25,000 identified phytochemicals have been shown to possess potent anticancer activities [72, 73]. The aerial parts of graviola have been extensively studied with several reported in vitro and in vivo pharmacological activities, and have been shown to be effective in the management of several cancer types. The detailed molecular mechanisms of action of various graviola organs against various cancers are summarized in tabular format (Table 3 and Figure 3).

## 4. Cytotoxicity

There is no universal definition for the term “cytotoxic drug.” Nonetheless, this term is commonly used in a variety of regulations for pharmaceutical development and manufacturing of drugs [74]. Simply put, a cytotoxic drug is an agent that has destructive actions on cells, often implying that these cells are targeted for destruction [75], a concept that certainly applies to many antineoplastic drugs [75].

The major bioactive components that have been extracted from various *A. muricata*'s parts are known as annonaceous acetogenins (AGEs). These are derivatives of long-chain (C32 or C34) fatty acids derived from the polyketide pathway, reviewed in [76]. Many of these derivatives are reported to be selectively toxic to cancer cells, including multidrug-resistant cancer cell lines [77]. Annonaceous acetogenins induce cytotoxicity, at least in part, by inhibiting mitochondrial complex I, which is involved in oxidative phosphorylation and ATP synthesis [78]. As cancer cells have a higher demand for ATP than the normal cells, mitochondrial complex I inhibitors have potential in cancer therapy [79].

Purified AGEs, such as annocatacin (A or B) [80] or annocatalin [81], have been found to induce significant cytotoxicity in Hep G2 and Hep 2,2,15 hepatic cancer cells *in vitro* [82, 83]. In breast cancer, cytotoxicity can be induced in MCF-7 cells using any of the following purified AGEs: annomuricin A, B [17], C [18], or E [16]; muricatocin A, B, or C [47]; muricapentocin [16]; annomutacin [43]; annohexocin [34]; annopentocin A, B, or C [32]; murihexocin A, B [15], or C [47]; muricoreacin [47]; muricatacin [48]; isoannonacin [49]; isoannonacin-10-one [49]; goniothalamicin [49]; gigantetrocin [49] A or B [50], muricatetrocin A or B [17, 84], *cis*-annonacin; *cis*-annonacin-10-one; *cis*-goniothalamicin; arianacin; or javoricin [85]. In addition, synergistic therapeutic effects have been shown with the combination of AGEs. For example, cytotoxicity in breast cancer (Figure 2) has been observed using a combination of (2,4-*cis*)-10R-annonacin-A-one and (2,4-*trans*)-10R-Annonacin-A-one [43], or a mixture of *cis*-annomuricin-D-one and *trans*-annomuricin-D-one [32]. Moreover, AGEs induce cytotoxicity in a variety of other cancers such as prostate [15, 16, 21, 22, 34, 47], colorectal [16, 17, 25, 32, 34, 44, 86], lung [15–17, 32, 34, 44, 47]^5^, leukemia [46], renal [15, 16, 32, 34, 47], pancreatic [87, 88], hepatic [35, 36], and oral [32, 43] cancers. Combinations of AGEs also exhibited cytotoxicity in colorectal (HT-29), lung (A549) [32], prostate (PC-3), renal (A498), and pancreatic (PACA-2) cancers [28].

Organic solvent extracts derived from the different parts of *A. muricata* (presumably containing multiple bioactive compounds) have also been shown to induce cytotoxicity in a variety of cancer cell lines. For example, leaf extracts induced cytotoxicity in human A375 melanoma [36], immortalized HaCaT keratinocytes, and MDA-MB-435S, previously cross-contaminated and mislabeled as breast carcinoma cells [89], but currently identified and authenticated as a melanoma cell line (M14) [56, 90], or head and neck squamous cell SCC-25 carcinoma [91], pancreatic (CD18/HPAF and FG/COLO357) [28], colorectal (HT-29 and HCT-116) [25], *Liver HepG2* [56], and lung A549 [31] cancer cell lines. Leaf extracts have also demonstrated reduced cell viability in pancreatic Capan-1 cancer cells [92]. Extracts derived from seeds are toxic to hepatic Hep G2 [31] cancer cells, while extracts from leaf, pericarp, seed, and stem have each shown cytotoxicity towards hematological malignant cells such as the leukemia U-937 cell line [56, 69].

The most commonly used solvents for the *A. muricata* extracts that induced cytotoxicity against cancer cells are ethanol and methanol (Table 3). Ethanolic leaf extracts induce cytotoxicity in breast MCF-7 [45] and MDA [93], colorectal COLO-205 and DLD-1 [94], lung H-460 [95], leukemic K562 [19] and ECV-304, also previously misidentified as a human umbilical vein endothelial cell line [96], but now re-authenticated as a T24-contaminated human bladder cancer cell line [90, 97, 98] and (see http://iclac.org/databases/cross-contaminations), stomach C-678 [95], melanoma A375 [36], and Ehrlich ascite EACC [93] cancer cells. However, according to available data, ethanolic fruit extracts induce toxicity only against both breast T47D [22] and lung H-460 [95] cancer cells. In addition, while ethanolic twig extract shows cytotoxic activity against ECV-304 cancer cells *in vitro* [96], methanolic extracts of the leaves, pericarp, or seeds of *A. muricata* all exert toxicity against glioma U87MG, breast MDA-MB-231-pcDNA3 and MDA-MB-231-*BCRP* clone 23, colorectal HCT-116 (*p53*^+*/*+^), and HCT-116 (*p53*^−*/*−^) cancer cells [24]. Moreover, stem methanolic extract induces cytotoxicity towards leukemia U-937 cells [33].

## 5. Apoptosis

Apoptosis, or programmed cell death, is integral for normal development and tissue homeostasis in most multicellular organisms [99]. Apoptosis plays a vital role in destroying cells which are selectively unnecessary or that present a threat to the integrity of an organism, thereby limiting the development and/or spread of cancer [1]. In many cancers, however, the gene(s) regulating apoptosis are faulty which leads to uncontrolled proliferation [100]. The ability to induce cellular apoptosis in tumor tissue is the key to finding a successful natural product as an anticancer agent [27, 101].

Apoptosis displays characteristic morphological and biochemical changes which may include cell shrinkage, nuclear fragmentation, chromatin condensation, and membrane blebbing [99, 102, 103]. The major apoptotic pathways are intrinsic and extrinsic [104]. The intrinsic (or mitochondrial) pathway can be induced through intracellular stresses such as DNA damage or oxidative stress leading to the release of mitochondrial cytochrome c forming the apoptosome complex [105]. This complex is composed of cytochrome c, apoptotic protease activating factor, and procaspase [106], which activates different caspases [107]. The extrinsic pathway, also known as the death receptor pathway, can be induced by death ligands, tumor necrosis factor *α*, and tumor necrosis factor-related apoptosis inducing ligand (TRAIL)) [108]. These ligands bind to their cell surface receptors (tumor necrosis factors), death receptors, and Fas causing sequential activation of caspase-8, caspase-3, and caspase-7 [109]. Moreover, apoptosis is regulated by several proteins such as BCL-2 [110], BAX [111], and PCNA [25].

Several studies examining the anticancerous properties in *A. muricata* extracts have observed the induction of apoptosis. According to the available data, there are about six extract types with regard to solvent extraction of *A. muricata* parts, including water [21], ethanol [20, 112], methanol, ethyl acetate [31], chloroform, and n-hexane [37] extracts. Leaf extracts of *A. muricata* induce apoptosis in breast MDA-MB-468 cancer cells through caspase-3 activation [23]. Similarly, *A. muricata* fruit extract induces apoptosis in breast T47D cancer cells [22]. An ethanolic extract of *A. muricata* leaves induces apoptosis [20] in COLO-205 colon cancer cells through upregulation of proapoptotic caspase-3 marker activity [112]. Similarly, in HT-29 colorectal cancer cells, annomuricin E derived from leaves of *A. muricata* induced apoptosis mediated through activation of caspases 3/7 and 9, upregulation of BAX, and downregulation of BCL-2 at the mRNA and protein levels [25]. In K562 leukemia cancer cells, an ethanolic leaf extract significantly enhanced caspase-3 activity to induce apoptosis, which was confirmed by a terminal deoxynucleotidyl transferase-mediated dUTP nick-end labelling (TUNEL) assay [19]. In addition, ethanolic extracts of roots, fruits, or leaves of *A. muricata* have been shown to induce apoptosis in HL-60 leukemia cancer cells through loss of MMP [27]. An aqueous leaf extract was shown to reduce prostate size which was suggested to have been due to apoptosis [21]. Leaf extracts prepared using various solvents were also able to induce apoptosis on HeLa cervical cancer cells [37]. Treatment of colorectal HT-29 and HCT-116 cancer cells with an ethyl acetate extract of leaves caused apoptosis through excessive accumulation of ROS followed by disruption of MMP, cytochrome c leakage, activation of the initiator and executioner caspases, upregulation of Bax, and downregulation of Bcl-2 protein [25]. An ethyl acetate *leaf* extract also elicited a 72.5% reduction in aberrant crypt foci inhibition in azoxymethane-induced colorectal carcinogenesis in rats [25]. This effect was associated with a downregulation of PCNA and Bcl-2 proteins and an upregulation of Bax protein as well as an increase in the levels of enzymatic antioxidants and a decrease in the malondialdehyde level of the colon tissue homogenates suggesting the suppression of lipid peroxidation [25]. Similarly, an ethyl acetate extract derived from leaves of *A. muricata* induced apoptosis in lung A549 cancer cells through elevating ROS formation, followed by attenuation of MMP via upregulation of Bax and downregulation of Bcl-2. These effects were accompanied by the release of cytochrome c into the cytosol, which triggered the activation of caspase-9 followed by caspase-3 activation. Moreover, the treatment also suppressed the induced translocation of NF-*κ*B from cytoplasm to nucleus [31]. Apoptosis also has been shown in HeLa cervical cancer cells after treatment with an ethyl acetate leaf extract [37]. Methanolic extracts of seeds or leaves were also shown to induce apoptosis in leukemic CCRF-CEM cells, while pericarp or leaf extracts induced apoptosis in CEM/ADR5000 leukemia cells [24]. Furthermore, leaf extracts using ethyl acetate and ethanol-distillate water, as well as *n*-hexane and chloroform leaf extracts, have also been shown to induce apoptosis in HeLa cervical cancer cells [37].

## 6. Modulation of Cellular Proliferation

Proliferation is a hallmark of cancer development and progression manifested by altered expression and/or activity of cell cycle-related proteins. In cancer, the normal cell cycle process is impaired, resulting in uncontrolled cell proliferation, growth, and tumor progression *A. muricata* extracts, and AGEs have been shown to regulate the cell cycle machinery, leading to cell cycle arrest and inhibition of cell proliferation. According to the available data, there are about seven AGEs and five extracts which have demonstrated antiproliferative activity. Specific AGEs include muricins J, K, L [29], M, and N; muricenin [30]; and annomuricin E [25]. Extracts with antiproliferative properties include those using solvents such as hexane [30], ethyl acetate [77], methanol [113], ethanol [27], and water [91] as discussed below.

Muricins J, K or L, M, and N and muricenin leaf AGEs all have shown antiproliferative activities when tested on human prostate PC-3 cancer cells [29, 30]. Annomuricin E derived from leaves of *A. muricata* was reported to suppress proliferation of HT-29 colorectal cancer cells via cell cycle arrest at the G1 phase, which also induces leakage of lactate dehydrogenase [25].

Hexane, ethyl acetate, and methanol extracts all significantly reduced cell proliferation in hepatic Hep G2 and breast MCF-7 and MDA-MB-231 cancer cells [4]. A hexane *leaf* extract significantly reduced cell proliferation in PC-3 colorectal cancer cells [30]. Hexane and methanol extracts of *A. muricata* suppressed the proliferation of HT-29 and HCT-116 colorectal adenocarcinoma cells [26] as well as A549 [4] lung cancer cells. Antiproliferative and cytotoxic activities were seen in NCI-H292 cancer lung cells after treatment with methanol pericarp extract [113]. Ethanol extracts of roots, fruits, or leaves inhibited proliferation via G0/G1 cell cycle arrest in leukemia HL-60 cancer cells [26]. Hexane or DMSO seed extracts inhibited cell proliferation in pancreatic cancer Capan-1 cells [92]. An aqueous *leaf* extract showed promising antiproliferative activity by arresting the cell cycle in the G2M phase in SCC-25 squamous cell carcinoma [91]. A methanol leaf extract inhibited proliferation of Hep-2 cancer cells, first reported as laryngeal cancer cell line [113], currently authenticated as a cross-contaminated HeLa cell line [90] (http://iclac.org/databases/cross-contaminations). Finally, *A. muricata* leaf extracts were reported to induce cell cycle arrest at the G0/G1 phase in MDA-MB-468 breast [23], HCT-116 [31], and HT-29 [25] colorectal and A549 [31] lung cancer cells, and our preliminary unpublished observations showed similar effects on two nonmelanoma skin cancer cell lines, namely a basal (UWBCC1) and a squamous (A431) carcinoma cell line [116].

## 7. Necrosis and Other Related Effects

The death of cells in a tissue due to chemotherapeutic agents is defined as “necrosis” which contributes to chemotherapy-induced cell death [115]. Necrotic cell death is distinguished from its counterpart, apoptosis, in that caspase activation is not required for cell death. Unlike apoptosis, chemotherapy-induced necrosis results in plasma membrane rupture, thus spilling the contents of the cell and triggering the immune system. This results in the inhibition of cellular metabolism and induces further necrosis through the downregulation of factors related to hypoxia and glycolysis (i.e., HIF-1*α*, NF-*κ*B, GLUT1, GLUT4, HKII, and LDHA) in pancreatic FG/COLO357 and CD18/HPAF cancer cells [28].

Cancer cell motility, migration, and invasion also play fundamental roles in cancer metastasis [116]. Therefore, inhibiting either cancer cell motility, migration, or invasion impedes metastasis, which is the cause of over 90% of patient deaths [117]. After treatment of pancreatic FG/COLO357 and CD18/HPAF cancer cells with leaf extracts, the motility of cancer cells was decreased [28]. More dramatically, treatment of HT-29 and HCT-116 colorectal cancer cells with an ethyl acetate leaf extract conspicuously blocked the migration and invasion of cancer cells [25, 26]. In addition to the preclinical studies with cell lines cited above, a subset of these effects have been demonstrated in a clinical model system [118].

## 8. Other Potential Health-Related Benefits

In addition to cancer chemopreventive and chemotherapeutic effects, graviola extracts and their constituents, individually or in combination, have shown therapeutic properties for other ailments that afflict humankind including chronic inflammatory and oxidative diseases, wounds, and noninfectious and infectious microbial and parasitic diseases. Graviola organs have been used as herbal medications against cystitis, diabetes, headaches, hypertension, insomnia, and liver diseases as well as antidysenteric, anti-inflammatory, and antispasmodic agents [119]. Other benefits thus far reported for graviola constituents, in addition to those listed above, have included anxiolytic, anticancer, antitumorigenic, antidepressant, gastroprotective, antimalarial, antinociceptive, immunomodulatory, antistress, and wound healing activities [4, 120], some of which are reviewed below.

## 9. Anti-inflammatory, Antinociceptive, Antiarthritic, Immunomodulatory, and Wound Healing Activities

Over the last few decades, herbal remedies and natural phytochemicals have garnered scientific interest for their utility in managing pain and inflammation. Natural agents including *A. muricata* can mechanistically modulate these effects by impacting molecular targets, some of which are common to pain medications such as NSAIDs, but with reduced side effects. The following examples from the literature are provided.

An ethanol extract of graviola leaves was administered orally to rats by de Sousa et al., followed by various tests of nociception and inflammation [119]. These authors found the following dose-dependent effects: (a) reduction in abdominal contortions after ip injection of acetic acid, (b) increased time to paw licking after subplantar injections of 2.5% formalin, (c) increased reaction time in a hot plate test, (d) reduced edema after subplantar injection of 2% carrageenan, and (d) reduced exudate and leucocyte counts after carrageenan-induced pleurisy. Taken together, these observations were interpreted by the authors as a confirmation of the ethnomedical use of ethanol extracts of graviola leaves for therapeutic purposes. However, one caution was that the ethanol extract was toxic to animals at approximately 1.7 g/kg, leading the authors to call for further studies to ensure safe usage in humans.

Similar tests with an ethanol extract of graviola leaves were carried out by [121], who confirmed the antinociceptive properties of this extract. These authors extended these studies to include an ethanol-induced ulcer model in rats pretreated with N-nitro-L-arginine methyl ester (L-NAME), finding that the graviola extract dose-dependently reduced the size of the ulcerative lesions. This effect was inhibited by N-ethylmaleimide, prompting the authors to conclude that the protective effect of the graviola extract in this setting might be due, at least in part, to antioxidant properties that increase the sulfhydryl content of the gastric mucosa [121].

Hamid et al. also tested an ethanol extract of graviola leaves, administered orally to rodents, for its acute and chronic anti-inflammatory actions. These authors reported that the ethanol extract (a) reduced xylene-induced ear edema in mice, (b) attenuated arthritis in rats induced by complete Freund's adjuvant, and (c) reduced TNF-*α* and IL-1*β* levels in the arthritis model, suggesting that the antiarthritic actions are at least partially due to a suppression of proinflammatory cytokines.

Ishola et al. used methodology similar to that described above (writhing, formalin, hot plate tests, carrageenan-induced rat paw edema, and xylene-induced ear edema tests) to test lyophilized fruit extracts of graviola [122]. These authors also found a dose-dependent inhibition of (a) writhes, (b) formalin-induced pain, (c) carrageenan-induced paw circumference, and (d) xylene-induced ear edema. This study also probed the possible involvement of the opiodergic, nitric oxide, and prostaglandin pathways, with the following results: (a) the anti-inflammatory actions of the fruit extracts were blocked by NG-nitro-L-arginine (a nitric oxide inhibitor) as well as by naloxone and (b) treatment with extract dose-dependently inhibited both COX-1 and COX-2. These authors therefore concluded that the analgesic and anti-inflammatory actions of a graviola fruit extract, as it is used in traditional African medicine, are confirmed and that these actions involve the opiodergic, prostaglandin, and nitric oxide systems [122].

Laksmitawati et al. tested an ethanol extract of graviola leaves in cultures of the LPS-stimulated murine macrophage cell line (RAW264.7) [123] and performed cell viability, cytokine ELISAs, and nitric oxide (NO) production assays. These authors reported that (a) cell viability was not affected in doses up to 50 *μ*g/mL and b) levels of TNF-*α*, IL-1*β*, IL-6, and NO were all reduced relative to untreated cells.

Oliveira et al. used an aqueous extract of graviola in a similar in vitro study of inflammatory markers using RAW264.7 cells along with cell-free assays in comparison with an aqueous extract from *Jasminum grandiflorium* [67]. In addition to examining NO production, these authors also performed HPLC with diode array detection (HPLC-DAD) to determine the phenolic compounds responsible for some of the effects. These authors reported that (a) graviola was superior to *J. grandiflorium* in inhibiting both NO production as well as phospholipase A_2_ (PLA_2_) and (b) aglycones from the extract especially quercetin and 5-O-caffeoylquinic acid were capable of inhibiting NO production and/or PLA_2_ in low micromolar concentrations. Cytotoxicity was not noted at the concentrations tested in this study [67].

Related to graviola's anti-inflammatory effects are its ability to promote wound healing; indeed, graviola preparations are commonly used in folk medicine for skin diseases and abscesses. Moghadamtousi et al. used an ethyl acetate extract of graviola leaves in an excisional wound rat model [4]. In addition to examining antioxidant activity of this extract (see discussion above), these authors also performed histological and immunohistochemical analysis of wounds treated with ointments containing the extract using intrasite gel as a positive control. Their results showed that (a) both doses of extract showed significant acceleration of wound closure and tissue regeneration, with the higher dose being comparable to the intrasite positive control, and (b) both doses of extract upregulated heat shock protein 70 (Hsp70) to levels comparable to those seen in the intrasite-positive control as monitored by immunohistochemical staining. These authors concluded that there is a clear wound healing effect of the extract, even though the bioactive compounds responsible for the effect have not yet been identified [4].

## 10. Antioxidant Activity

Several reports have described the antioxidant properties of various graviola-derived extracts. These will be described in order of appearance in the literature.

Given that graviola leaves are used in Cameroon to manage diabetes, Florence et al. tested an aqueous extract of graviola leaves in streptozotocin-induced diabetic rats [54]. Although this extract had no effect on normal rats, the aqueous extract was found to reduce blood glucose levels in diabetic rats. These authors also found the following: (a) after 15 days of treatment with 100 mg/kg of extract (given 3 days prior to streptozotocin), animals showed significant (46%) reductions in blood glucose compared to diabetic controls not treated with extract, (b) immunostaining at the end of treatment showed preservation of pancreatic *β*-cells in treated animals compared to diabetic controls not treated with extract, (c) activity of superoxide dismutase (SOD) and catalase in diabetic animals treated with extract (100 mg/kg) were normalized up to levels seen in nondiabetic controls, and (d) levels of tissue malondialdehyde (a marker of lipid peroxidation) and nitrites were reduced down to levels seen in nondiabetic animals. These studies in rats support the use of graviola as an antidiabetic agent and suggest that at least part of its beneficial actions are antioxidant in nature. One cautionary observation was made, however: a higher dose (200 mg/kg) of extract was not only less effective but also resulted in 25% mortality among that treatment group [54].

Gavamukulya et al. tested the antioxidant potential of ethanolic and aqueous extracts of graviola found in Eastern Uganda using 2,2-diphenyl-2-picrylhydrazyl (DPPH•) and reducing power assays [93]. Their results indicated that (a) the ethanolic extract was superior to the aqueous extract with respect to both reducing power and in vitro antioxidant activity and (b) the ethanolic extract, but not the aqueous extract, was selectively cytotoxic to three tumor cell lines as opposed to no effect on normal spleen cells [93].

George et al. compared methanolic and aqueous extracts of graviola with respect to their free radical scavenging and DNA protective properties using several assays including a ferric reducing antioxidant property (FRAP) assay, a DPPH• radical scavenging assay, a hydroxyl scavenging activity assay (HRSA), and a DNA damage protective activity [124]. These authors also carried out HPLC analysis of phenolic compounds in each extract. Both graviola extracts were found to possess significant radical scavenging assays, and a strong positive correlation was seen between the total phenolic content and the radical scavenging activity of each extract. The methanolic extract was found to confer superior protection against hydrogen peroxide-induced DNA damage [124].

Moghadamtousi et al. applied an ethyl acetate extract of graviola leaves to skin wounds in rats [4]. Although wound healing was the main focus of this study (see above), these authors also measured levels of malondialdehyde as well as activities of catalase, glutathione peroxidase, and superoxide dismutase in wound tissue homogenates. After 15 days of treatment, analysis of tissue samples revealed a “significant surge in antioxidants activities and decrease in the MDA level of wound tissues compared with vehicle control,” providing yet another example of the antioxidative potential of graviola extracts [124].

Finally, Son et al. studied the antioxidant properties of steam and 50% ethanol extracts of graviola leaves in HepG2 cells [125]. Their results, standardized in some cases to vitamin C equivalents, indicated that (a) the 50% ethanol extracts were superior than steam extracts in scavenging peroxy and nitrogen radicals, although both were effective, and (b) the 50% ethanol extract upregulated superoxide dismutase 1 (SOD1) and Nrf2 (an important transcriptional regulator of antioxidant enzymes), but not catalase or heme oxygenase 1 (HMOX1).

In summary, accumulating evidence suggests that components of graviola possess potent antioxidant properties, although these may vary depending on the method of extraction as well as the cells/tissues in which they are tested.

## 11. Hepatoprotective Effects Related to Antioxidation

As described above, Adewole and Ojewole observed hepatic benefits after administration of an aqueous leaf extract of graviola to streptozotocin-induced diabetic rats [39]. The described benefits in liver consisted mainly of increases in antioxidant enzymes (catalase, SOD, and glutathione peroxidase) and levels of glutathione to reduce oxidative stress in this tissue. However, other positive effects of this treatment included improvements in blood lipid levels, specifically a decline in diabetes-induced levels of LDL, total cholesterol, and triglycerides and an increase in HDL [39].

Padma et al. tested the ability of an ethanol extract of graviola stem bark, in comparison with a similar extract from *Polyalthia cerasoides*, to ameliorate carbon tetrachloride liver toxicity in albino rats [126], using liver function tests as a measure of liver damage. Their results showed that the graviola extract was superior to the *P. cerasoides* extract in (a) reducing blood levels of liver transaminases (ALT/SGPT and AST/SGOT) and alkaline phosphatase released into the blood by the liver damage and (b) reducing lipid peroxidation in liver samples as measured by detection of MDA. While the protection of liver was not complete, the reduction in these measures of liver damage by graviola (but not by *P. cerasoides*) was highly significant (*P* < 0.001) in each case when compared to controls (number of animals in each group ranged from 7 to 10).

## 12. Antidiabetic and Hypotensive Properties

As already mentioned, graviola extracts have been used as antidiabetic agents in many parts of the world (see [127] above). Additional evidence for the presence of antidiabetic agents in graviola extracts has been reported as follows.

Adewole and Ojewole administered an aqueous leaf extract of graviola to streptozotocin-induced diabetic rats [39]. After four weeks of treatment, these researchers found that (a) glucose levels were reduced in treated diabetic rats along with an elevation in blood insulin, (b) levels of ROS and blood lipids also declined in treated diabetic rats, and (c) activities of antioxidant enzymes (catalase, SOD, and glutathione peroxidase) and levels of glutathione were increased in liver relative to untreated diabetic animals. This study mainly focused on the hepatic benefits of graviola treatment in the setting of diabetes, with the conclusion that graviola is protective against oxidative stress in this tissue.

In addition to antidiabetic properties of graviola (*Annona muricata*), it should also be noted that a closely related plant, *Annona montana* (also known as “false graviola”), has been tested for its effect on blood lipids and blood glucose. Treatment of Wistar rats for 40 days with juices prepared from either leaf or fruit not only showed reduced blood glucose and blood low-density lipoproteins but also increased levels of high-density lipoproteins, prompting these authors to propose that use of this plant may help prevent diabetes mellitus and dyslipidemia [128].

Finally, Nwokocha et al. administered an aqueous leaf extract by IV injection to normotensive Sprague-Dawley rats, and short-term measurements (10 minutes apart) were taken of systolic and diastolic blood pressure, after which animals were sacrificed and aortic rings were isolated and tested in an isometric force transducer system [12]. These authors reported that (a) both systolic and diastolic blood pressures were reduced in a dose-dependent manner by the graviola extract, but without an effect on heart rate; (b) further tests determined that these effects were apparently not due to muscarinic, endothelial, histaminergic, or adrenergic mechanisms; and (c) testing of aortic rings suggested that alkaloid components of the extract might be exerting these effects via blockade of calcium ion channels, speculating that reticuline, an alkaloid found in graviola leaves [129], might be contributing to these effects [12].

## 13. Antimicrobial and Antiparasitic Potentials

Graviola parts have been used as antimicrobial and antiparasitic agents in traditional medicine. Examples are provided according to the year published, except for antimalarials which will be discussed at the end of this section.

Bories et al. studied methanol extracts of two species of *Annona*, including muricata (graviola) and cherimolia, for their ability to inhibit growth of the parasites *Entamoeba histolytica*, *Trichomonas vaginalis*, *Nippostrongylus brasiliensis*, *Molinema dessetae*, and *Artemia salina* [130]. Results were expressed in terms of minimum inhibitory concentration (MIC), compared to reference compounds metronidazole (for *E. histolytica*) and ivermectin (for *N. brasiliensis* and *M. dessetae*). The results with crude extracts showed that (a) the *A. cherimolia* extract performed better overall in these tests and (b) both extracts showed only weak activity against the protozoans *E. histolytica* and *T. vaginalis* but strongly inhibited the *M. dessetae* filaria larvae, although at doses still an order of magnitude or so higher than the reference ivermectin compound. Nevertheless, this last result prompted the authors to perform fractionation to identify compounds with effectiveness against *M. dessetae*, leading to the identification of seven acetogenins with filaricidal activity, four of which displayed LD_50_ values equal to or lower than the ivermectin reference [130].

Osorio et al. compared 36 different extracts from six different plants, including six extracts from graviola, namely, hexane, ethyl acetate, and methanol extracts from stem and from leaves, for their effects on three *Leishmania* species, as well as on *Trypanosoma cruzi* and *Plasmodium falciparum* [131]. Briefly summarized, an ethyl acetate extract of graviola leaves and stems was found to have potent activity against *Leishmania*, but the relatively high toxicity displayed by the leaf extract in cultures of U-937 cells led the authors to conclude that the effectiveness against the *Leishmania* parasite likely reflected its toxicity against the host mammalian cells [131].

Vila-Nova et al. tested specific compounds from both graviola and *Platymiscium floribundum* for their effectiveness against three *Leishmania* species (*donovani*, *mexicana*, and *major*) [132]. The most potent leishmanicidal compound against all three species was the acetogenin annonacinone (EC_50_ approximately 7 *μ*g/mL), although corossolone and the coumarin scoparone (the latter compound from *P. floribundum*) also displayed moderate activity, suggesting that these compounds merit further development for treatment of leishmaniasis [132].

Mathew et al. examined aqueous extracts of leaves from graviola and *Simarouba glauca* on the dental root canal pathogen *Enterococcus faecalis* [133]. These authors reported that the graviola extract, but not the *S. glauca* extract, was as effective against *E. faecalis* in vitro as the sodium hypochlorite (1% bleach) reference disinfectant, providing a possible alternative for root canal irrigation.

Mithun Pai et al. similarly tested an aqueous extract of graviola leaves against the oral pathogens *Streptococcus mutans*, *Streptococcus mitis*, *Porphyromonas gingivalis*, *Prevotella intermedia*, and *Candida albicans* using an agar disc method [66]. Their results showed that (a) graviola displayed effectiveness against all organisms except for *P. intermedia*, with the highest dose being the most effective and a trend toward dose dependence, and (b) compared to the “gold standard controls” of ciprofloxacin for the bacteria and fluconazole for the *Candida* yeast, the graviola extracts were far less effective, but may warrant further testing especially in view of (to paraphrase the authors) their combined antimicrobial and (reputed) anticancer effects [66].

Simo et al. prepared ethanol extracts of leaves, stems, and roots from several plants belonging to the Annonaceae family, including graviola, and tested them for phenolic and flavonoid content, as well as antioxidant and antifungal activities, using various strains of *Candida* yeast and *Cryptococcus neoformans* [134]. Briefly summarizing the results, these authors showed that (a) all three extracts from graviola possessed free radical-scavenging activity (root being highest) and (b) all three compounds showed “moderate” antifungal activity (approximately two orders of magnitude less potent on a mg/mL basis as compared with a nystatin control).

Several studies have investigated the ability of extracts from plants, including graviola, to treat malaria caused by *Plasmodium falciparum*, with particular interest in chloroquine-resistant strains of this organism. Ménan et al. prepared aqueous, ethanol, and pentane extracts of 18 plants used in traditional medicine, including graviola leaves, and tested these in culture against two African strains of *P. falciparum*, one sensitive to chloroquine and the other resistant to the drug, to determine IC_50_ values (defined as concentrations required to inhibit *P. falciparum* growth in human blood cell culture by 50%) [36]. These authors also tested cytotoxicity of each extract on A375 melanoma cells, using incorporation of radiolabeled hypoxanthine as a measure of cell growth. Their results showed that (a) IC_50_ values in most cases were highest for the pentane extracts, with the graviola pentane extract showing not only “excellent” antiplasmodial activity but also a favorable ratio of antiplasmodial to cytotoxicity activity (ratio greater than 10), (b) activity against the chloroquine-resistant strain was just as good, if not better, than activity against the chloroquine-sensitive strain, and (c) in contrast to the antiplasmodial activities, the cytotoxic effects were greatest with the ethanol extract (6–7 fold lower IC_50_ compared with the pentane extract; the aqueous extract was largely ineffective in this assay). It should be noted that extracts from *Uvaria afzelii* and *Cola caricaefolia* also displayed significant antiplasmodial activities [36].

Mohd Abd Razak et al. tested the antiplasmodial activity of 54 extracts from 14 medicinal plant species using an ELISA assay for *P. falciparum* histidine-rich protein; cytotoxicity was evaluated in Madin-Darby bovine kidney (MDBK) cells using an MTT assay [135]. These authors reported that 11 of these extracts possessed antiplasmodial activities with “negligible” toxicity (ratio of antiplasmodial to cytotoxicity activity greater than 10). Specific findings for graviola were that all three leaf extracts tested (aqueous, methanol, and dichloromethane) displayed “promising” EC50 values of between 0.1 and 1 *μ*g/mL combined with low toxicity to MDBK cells, with a ratio of antiplasmodial to cytotoxicity activity of >750 for the aqueous extract [135].

Somsak et al. also tested an aqueous leaf extract from graviola for its antiplasmodial activity and acute toxicity using an in vivo, *Plasmodium berghei*-infected mouse model [13]. To test for antimalarial action, mice were injected ip with parasitized erythrocytes, followed by four consecutive days of oral treatment with 100–1000 mg/kg of extract, using chloroquine as a positive control. The results indicated a significant and dose-dependent inhibition of parasitemia (up to over 85% at the highest dose), along with prolonged survival from 7 days in untreated mice up to almost 29 days in mice treated with the highest dose, almost equaling the effectiveness of chloroquine (99% inhibition of parasitemia and 30-day survival). Toxicity studies showed no mortality doses up to 4000 mg/kg. The authors concluded that graviola extracts could potentially be the basis for the development of safe, effective, and affordable antimalarial agents [13].

Yamthe et al. tested a series of extractions and subfractions from both *A. muricata* (graviola) and the related *A. reticulata* for their activity against *P. falciparum* (strain W2 in culture) as well as cytotoxicity against human erythrocytes and foreskin fibroblasts [136]. Whereas all extracts showed low toxicity and some ability to inhibit growth of *P. falciparum*, thus supporting traditional use of these plants against malaria, the most potent extract was found by these authors to be a column chromatography subfraction from a methylene chloride stem bark extract of graviola, which displayed and IC_50_ of 70 ng/mL, with a ratio of antiplasmodial to cytotoxicity activity of >140. The authors planned to continue characterizing active subfractions and individual compounds to improve antiplasmodial activity and minimize cytotoxicity [136].

## 14. Toxicological and Safety Information

As briefly discussed above, several studies have included cytotoxic assays and other means of assessing toxicity in their studies of graviola extracts. The following comments are intended to provide perspective into the possibility of toxicity in the use of graviola components. Caparros-Lefebvre and Elbaz in 1999 reported that consumption of teas and fruits of some tropical plants, including graviola, was associated with atypical parkinsonism, leading to speculation that graviola might contain neurotoxins reviewed by Gavamukulya et al. [120]. No safety studies have been found to assess the efficacy of the extracts of *A. muricata* on the various cancers. A study discussed the possible connection between tropical fruit intake and the occurrence of atypical parkinsonism in the French West Indies [4, 120]. Another study in Guadeloupe Island publicized an association between intake of AGEs and the endemicity of a neurodegenerative disease [137], suggesting that AGEs are environmental neurotoxins accountable for neurodegenerative disorders. One of several follow-up studies focused on one of the AGE compounds, namely, annonacin, in causing tau-related neuropathology [137]. However, a consensus was reached in 2010 that consumption of species of Annonaceae was not directly related to occurrence of atypical parkinsonism (reviewed in [4, 120]). Other toxicologic findings discussed above in relation to the individual studies nevertheless merit serious consideration such that future studies of the use of graviola components must include rigorous safety testing since the content of potential toxins could vary according to the part of the plant, the extraction method, the location where the plant is grown, and even the time of harvest. Employing mesencephalic dopaminergic neurons, rat striatal neuronal cells, and laboratory rats, the neurotoxicity of seven acetogenins was evaluated, and the most abundant acetogenin (annonacin) and alkaloid (reticuline) from *A. muricata* were demonstrated to be neurotoxic [4]. Annonacin is a thousand times (1000x) more toxic to cultured neuronal cells than reticuline and a hundred times (100x) more potent than 1-methyl-4-phenylpyridinium, a known neurotoxin that effects parkinsonism in humans and animal models. Intravenous administration of isolated annonacin to laboratory rats was determined to estimate the amount of annonacin a human should consume via the ingestion of fruit daily for one year. In this regard, AVIS (l'Agence Francaise de Sécurité des Aliments) issued a conclusive statement that based on available data, it is not possible to link atypical parkinsonian syndrome cases identified in Guadeloupe to consumption of plan species belonging to the Annonaceae family (reviewed in [4]).

## 15. Conclusion and Future Prospects

Despite enhanced synthetic small molecule-based targeted anticancer therapies with improved patient prognosis, cancer remains a leading cause of death worldwide, as a result of challenges including increased toxicity and development of resistance to treatment agents. Natural products found in medicinal plants have great promise for the treatment of cancer [71]. This current review demonstrates *Annona muricata*'s anticancer potential and other health-related benefits by providing insights into its bioactive chemical constituents as well as the in vitro and in vivo studies that have been carried out in order to elucidate the molecular mechanisms of action of these constituents. Graviola not only is a sought-after tropical tree plant as an important foundation for the food industry and alternative traditional medicine but is also endowed with a wealth of phytochemicals with a wide variety of biological activities including its most prominent anticancer, antioxidant, and other properties not limited to those discussed herein.

Acetogenins and other secondary metabolites, including alkaloids, of this plant possess demonstrable ability to decrease growth of cancer that could be further comprehensively exploited. Acetogenins or other *A. muricata*-derived compounds could be tested as monotherapy or as sensitizers in combination with standard cancer treatments for cancer patients. Numerous studies have reported anticancer actions of *A. muricata*. However, more rigorous evaluation of various plant parts, their extracts, and ultimately isolated bioactive compounds is clearly warranted. Indeed, the most effective bioactive compounds from *A. muricata* could potentially serve as scaffold entities for design and synthesis of derivatives that may even be more efficacious in preclinical and clinical trials. Whereas earlier studies characterized the biological activities of extracts from different graviola organs, future studies pivotal for the development of pharmaceutical and agricultural product development should focus on investigating on the biochemical and physiological functions of active phytochemical compounds and their combinations, as well as the detailed molecular mechanisms causal to these activities, progressing from in vitro studies to in vivo rodent models and ultimately to clinical trials to assess safety as well as therapeutic efficacy of the most promising graviola components.

There is already one clinical case report which describes a 66-year-old female who was diagnosed with metastatic breast cancer and whose metastases had progressed even after multiple rounds of chemotherapy including anthracyclines and taxanes. This patient self-medicated by boiling 10–12 dry leaves of *A. muricata* in water for 5–7 minutes, then orally consumed an 8 oz daily dose of this aqueous extract. Her metastases remained stable for 5 years on graviola (together with Xeloda) [118]. This report, although anecdotal, hints of the untapped antimetastatic potential of *A. muricata* and its components.

In a rodent in vivo study, graviola leaf extract inhibited 59.8% of pancreatic cancer growth of cells and their metastasis induced by CD18/HPAF cells in a mouse model [28].

In order to exploit the full medicinal potential of *A. muricata*, existing gaps in our knowledge of annonaceous acetogenins and other bioactive compounds should be addressed. Currently, there are no target-based approaches for evaluating components derived from *A. muricata* in cancer therapy. With the rapidly expanding knowledge of the pathways and networks that control cell signaling, proliferation, metastasis, and cell death, the possibility of using these components in a targeted fashion is very appealing in order to expand our anticancer armamentarium. Numerous in vitro and preclinical in vivo studies have supported most of the traditionally acclaimed benefits, but these must be validated in human clinical trials. The more than 200 phytochemicals identified in graviola mainly including acetogenins, alkaloids, and phenols have shown a multitude of pharmacological activities as discussed herein, and it is hoped that future studies will identify novel phytochemical scaffolding entities which are yet to be identified from *A. muricata*.

While most have provided health benefits, some of the derived phytochemicals like acetogenins have demonstrated in vitro and in vivo neurotoxicity. Although current consumption does necessarily lead to acute toxicity, further research to identify and quantify the amount of toxic phytochemicals as well as determine the doses to be exposed to humans to induce toxicity is urgently needed. For future directions and safety concerns in developing these plant aerial parts and their constituents, current studies indicate that phytochemical composition and anticancer properties vary with the geographical sources of *A. muricata* [58]. Because consistent results are difficult to achieve since processing and formulation may vary from the desired biological activity, there is therefore the need for systematic and robust screening to identify biochemical fractions as well as the physiological effects of all isolated constituents by detailing the investigation of underlying mechanisms. More importantly, for more safety, the toxicological profile is required to be documented [137, 138]. Another area previously neglected that needs to be intensely focused on is clinical trials concerning the rich pharmaceutical potential of *A. muricata*.

In conclusion, the authors intended this review to be a call to action for the development of better graviola-based pharmaceutical, agricultural, and food industrial agents, for the human diseases and conditions discussed herein, and possibly for other conditions for which graviola components have not even been tested. Given that graviola is already widely used in traditional medicine, these agents, if properly tested and produced, could potentially represent huge benefit by providing accessible and affordable agents against many of the conditions that plague humankind.

## Figures and Tables

**Table 1:** Different solvent extracts of *A. muricata* and their reported anticancer activities.

Extract (solvent)	Cancers (cell lines)
*n*-Hexane	Cervical (HeLa) cancer [37]
Chloroform	Cervical (HeLa) cancer [37]
Pentane	Melanoma (A375) cancer [36]
*n*-Butanolic	(MDA-MB-435S) cancer [89], now known as a melanoma cell line [90]
DMSO	Pancreatic (Capan-1 [92], FG/COLO357, and CD18/HPAF [28]) cancer
Fungal strain	Breast (MCF-7) [38], colorectal (HTC-8) [38], lung cancer (A549) [38], hepatic (Bel-7402) [38], gastric (BGC-823) [38], and ovarian (A2780) [38] cancers
H_2_O	Squamous cell carcinoma (SCC-25) [91], melanoma (A375) [36], prostate (PC-3) [21], pancreatic (CD18/HPAF) [28], and breast cancer patients [118]
Hexane	Breast (MCF-7 and MDA-MB-231) [31], colorectal (HT-29 and HCT-116) [26], lung cancer (A549) [31], leukemic (U-937) [33, 46], pancreatic (Capan-1) [92], and hepatic (Hep G2) [31] cancers
Ethyl acetate	Breast (MCF-7 and MDA-MB-231) [31], colorectal (HT-29 and HCT-116) [25], lung (A549) [31], leukemic (U-937) [33, 46, 131], hepatic (Hep G2) [31], and cervical (HeLa) [37] cancers.
Ethanol	Ehrlich ascite carcinoma (EACC) [93], breast (MCF-7 [45], MDA-MB-231-BCRP clone 23 [77, 139], T47D [22], MDA and SKBR3 [93]), colorectal [20] [140] (COLO-205 and DLD-1) [94], lung (H-460) [45, 95], leukemic (K562 [19] [96], ECV304 [96] and HL-60 [27]), stomach (C-678) [95], melanoma (A375) [36], skin [141], glioma (SF-268) [45], and cervical (HeLa) [37] cancers
Methanol	Breast (MCF-7 and MDA-MB-231 [31], MDA-MB-231-pcDNA3, and MDA-MB-231-BCRP clone 23 [24]), colorectal (HT-29 and HCT-116 [26], HCT116 (*p53*^+*/*+^), and HCT116 (*p53*^−*/*−^) [24]), lung (A549 [31] and NCI-H292 [113]), leukemic (U-937 [33, 46], CCRF-CEM, and CEM/ADR5000 [24]), hepatic (Hep G2 [24, 31] and Hep 2,2,15 [31]), glioma (U87MG and U87MG.*ΔEGFR*) [24], and laryngeal (currently cervical HeLa; Hep-2) [113] cancers

**Table 2:** AGEs of *A. muricata* reported to have anticancer activities. Structures were drawn using ChemDraw, Arial, point 20.

Compound names	Structure	Molecular formula	MWT (g/mol)	Cancer cell lines tested on
Annocatacin A	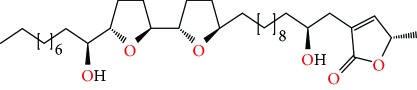	C_35_H_62_O_6_	578.88	Hepatic (Hep G2 and Hep 2,2,15) cancer [80]
Annocatacin B	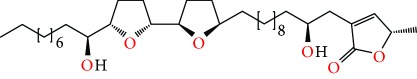	C_35_H_62_O_6_	578.88	Hepatic (Hep G2 and Hep 2,2,15) cancer [80]
Annocatalin	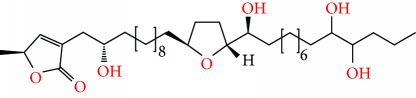	C_35_H_64_O_7_	596.89	Hepatic (Hep G2 and Hep 2,2,15) cancer [81]
Annohexocin	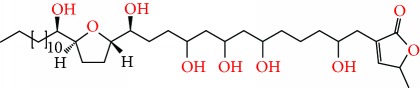	C_35_H_64_O_9_	628.888	Breast (MCF-7), prostate (PC-3), colorectal (HT-29), lung (A549), renal (A498) and pancreatic (PACA-2) cancers [34]
Annomuricin A	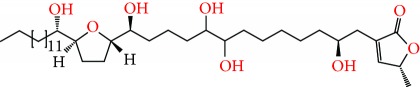	C_35_H_64_O_8_	612.889	Breast (MCF-7), colorectal (HT-29), lung (A549) [17], and leukemic (U-937) [46] cancers
Annomuricin B	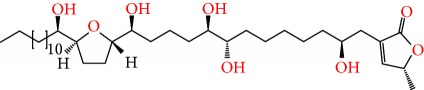	C_35_H_64_O_8_	612.889	Breast (MCF-7), colorectal (HT-29), and lung (A549) cancers [17]
Annomuricin C	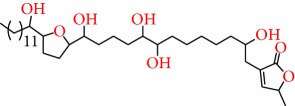	C_35_H_64_O_8_	612.889	Breast (MCF-7), colorectal (HT-29), and lung (A549) cancers [86]
Annomuricin E	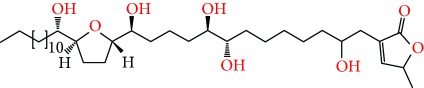	C_35_H_64_O_8_	612.889	Breast (MCF-7), prostate (PC-3), lung (A549), renal (A498), pancreatic (PACA) [16], and colorectal (HT-29) cancers [16, 25]
Annomutacin	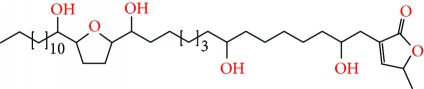	C_37_H_68_O_7_	624.944	Breast cancer (MCF-7), colorectal (HT-29), and lung cancers (A549) [43]
Annonacin	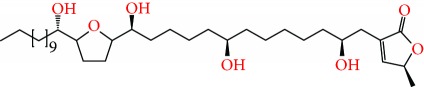	C_35_H_64_O_7_	596.89	
Annonacin A	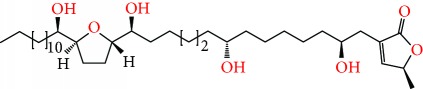	C_35_H_64_O_7_	596.89	Leukemia (U-937) [46]
Annonacinone	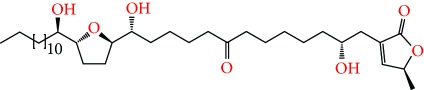	C_35_H_62_O_7_	594.874	Oral cancer (KB) [35]
Annopentocin A	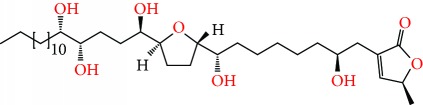	C_35_H_64_O_8_	612.889	Breast (MCF-7), prostate (PC-3), colorectal (HT-29), lung (A549), renal (A498), and pancreatic (PACA-2) cancers [32]
Annopentocin B	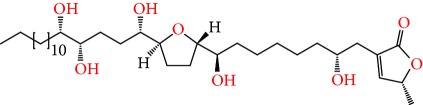	C_35_H_64_O_8_	612.889	Breast (MCF-7), prostate (PC-3), colorectal (HT-29), lung (A549), renal (A498), and pancreatic (PACA-2) cancers [32]
Annopentocin C	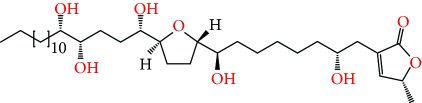	C_35_H_64_O_8_	612.889	Breast (MCF-7), prostate (PC-3), colorectal (HT-29), lung (A549), renal (A498), and pancreatic (PACA-2) cancers [32]
Arianacin	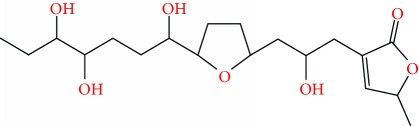	C_35_H_64_O_7_	596.89	Breast (MCF-7), colorectal (HT-29), and lung (A549) cancers [85]
Corossolin	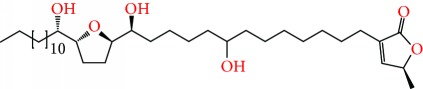	C_35_H_64_O_6_	580.891	Hepatic (Hep G2 and Hep 2,2,15) [82] and oral (KB) cancers [35]
Corossolone	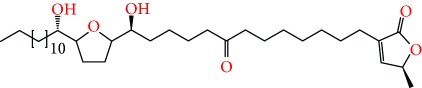	C_35_H_62_O_6_	578.875	Hepatic cancer (Hep G2 and Hep 2,2,15) [82] and oral (KB) cancers [35, 83]
Gigantetrocin	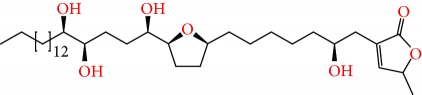	C_35_H_64_O_7_	596.89	Breast (MCF-7), colorectal (HT-29), and lung (A549) cancers [49]
Gigantetrocin A	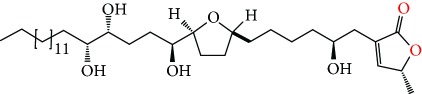	C_35_H_64_O_7_	596.89	Breast (MCF-7), colorectal (HT-29), and lung (A549) cancers [50]
Gigantetrocin B	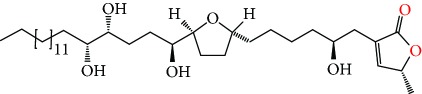	C_35_H_64_O_7_	596.89	Breast (MCF-7), colorectal (HT-29), and lung (A549) cancers [17, 50]
Goniothalamicin	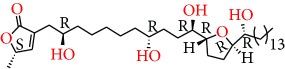	C_35_H_64_O_7_	596.89	Breast (MCF-7), colorectal (HT-29), and lung (A549) cancers [49]
Isoannonacin	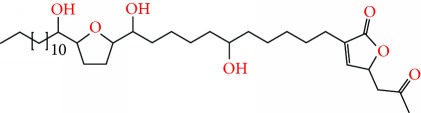	C_35_H_64_O_7_	596.89	Breast (MCF-7), colorectal (HT-29), and lung (A549) cancers [48]
Isoannonacin-10-one	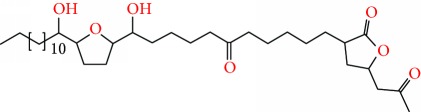	C_35_H_62_O_7_	594.874	Breast (MCF-7), colorectal (HT-29), and lung (A549) cancers [49]
Javoricin	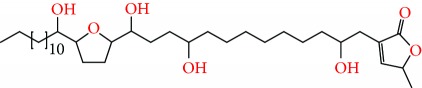	C_35_H_64_O_7_	596.89	Breast (MCF-7), colorectal (HT-29), and lung (A549) cancers [85]
Longifolicin	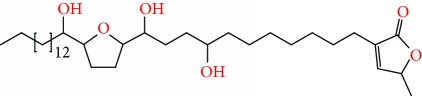	C_35_H_64_O_6_	580.891	Hepatic (Hep G2 and Hep 2,2,15) cancer [82]
Muricapentocin	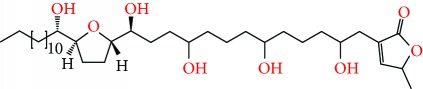	C_35_H_64_O_8_	612.889	Breast (MCF-7), prostate (PC-3), colorectal (HT-29), lung cancer (A549), renal (A498), and pancreatic (PACA) cancers [16]
Muricatacin	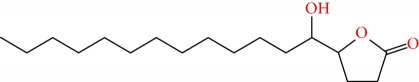	C_17_H_32_O_3_	284.44	Breast (MCF-7), colorectal (HT-29), and lung (A549) cancers [48]
Muricatetrocin A	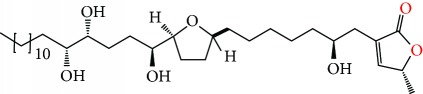	C_35_H_64_O_7_	596.89	Breast (MCF-7), colorectal (HT-29), and lung (A549) cancers [17, 50]
Muricatetrocin B	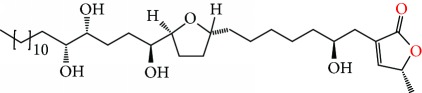	C_35_H_64_O_7_	596.89	Breast (MCF-7), colorectal (HT-29), and lung (A549) cancers [17, 50]
Muricatocin A	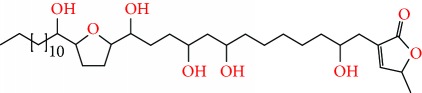	C_35_H_64_O_8_	612.889	Breast (MCF-7), colorectal (HT-29) and lung (A549) cancers [44]
Muricatocin B	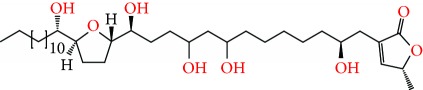	C_35_H_64_O_8_	612.889	Breast (MCF-7), colorectal (HT-29), and lung (A549) cancers [44]
Muricatocin C	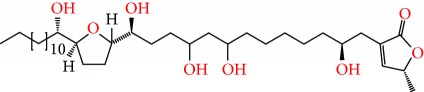	C_35_H_64_O_8_	612.889	Breast (MCF-7), colorectal (HT-29), and lung (A549) cancers [86]
Muricenin	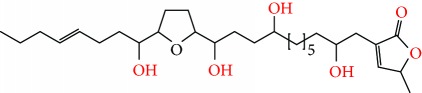			Prostate (PC-3) cancer [30]
Muricin A	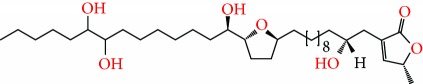	C_35_H_64_O_7_	596.878	Hepatic (Hep G2 and Hep 2,2,15) cancer [82]
Muricin B	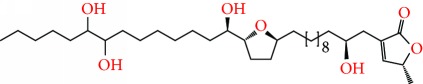	C_35_H_64_O_7_	596.878	Hepatic (Hep G2 and Hep 2,2,15) cancer [82]
Muricin C	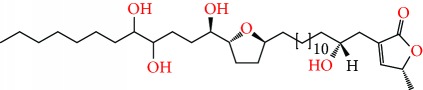	C_35_H_64_O_7_	596.878	Hepatic (Hep G2 and Hep 2,2,15) cancer [82]
Muricin D	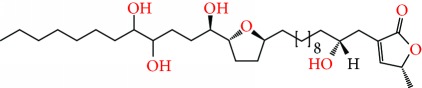	C_33_H_60_O_7_	568.836	Hepatic (Hep G2 and Hep 2,2,15) cancer [82]
Muricin E	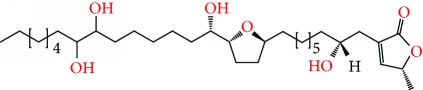			Hepatic (Hep G2 and Hep 2,2,15) cancer [82]
Muricin F	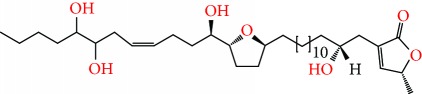	C_35_H_62_O_7_	594.874	Hepatic (Hep G2 and Hep 2,2,15) cancer [82]
Muricin G	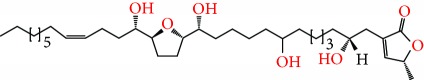			Hepatic (Hep G2 and Hep 2,2,15) cancer [82]
Muricin H	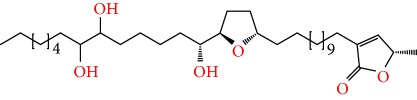	C_35_H_64_O_6_	580.891	Hepatic (Hep G2 and Hep 2,2,15) cancer [81]
Muricin I	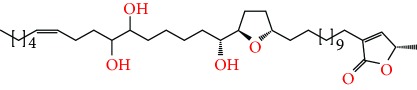	C_37_H_66_O_6_	606.929	Hepatic (Hep G2 and Hep 2,2,15) cancer [81]
Muricin J	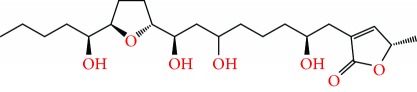	C_22_H_38_O_7_	414.2618	Prostate (PC-3) cancer [29]
Muricin K	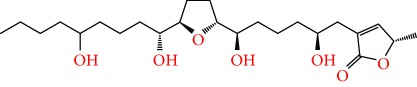	C_24_H_42_O_7_	442.2931	Prostate (PC-3) cancer [29]
Muricin L	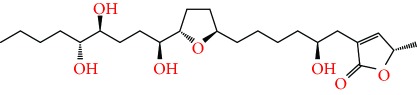	C_24_H_42_O_7_	442.2931	Prostate (PC-3) cancer [29]
Muricin M	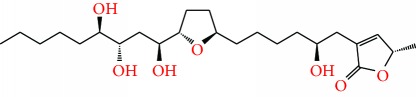	C_24_H_42_O_7_	442.2931	Prostate (PC-3) cancer [30]
Muricin N	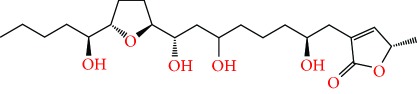	C_22_H_38_O_7_	414.2618	Prostate (PC-3) cancer [30]
Muricoreacin	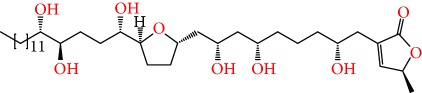	C_35_H_64_O_9_	628.4550	Breast (MCF-7), prostate (PC-3), colorectal (HT-29), lung cancer (A549), renal cancer (A498), and pancreatic (PACA-2) cancers [47]
Murihexocin A	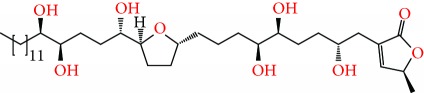	C_35_H_64_O_9_	628.888	Breast (MCF-7), prostate (PC-3), colorectal (HT-29), lung (A549), renal (A498), and pancreatic (PACA-2) cancers [15]
Murihexocin B	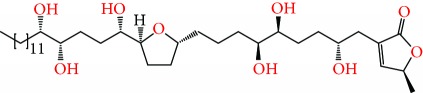	C_35_H_64_O_9_	628.4550	Breast (MCF-7), prostate (PC-3), colorectal (HT-29), lung (A549), renal (A498), and pancreatic (PACA-2) cancers [15]
Murihexocin C	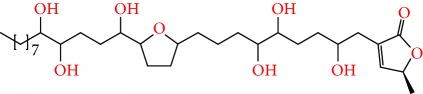	C_31_H_56_O_9_	572.3924	Breast (MCF-7), prostate (PC-3), colorectal (HT-29), lung cancer (A549), renal cancer (A498), and pancreatic (PACA-2) cancers [47]
Murisolin	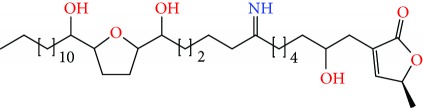	C_35_H_63_NO_6_	593.4655	Oral (KB) cancer [35]
Solamin	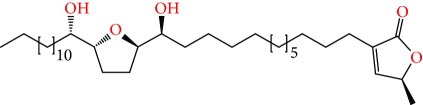	C_35_H_64_O_5_	564.892	Oral (KB) cancer [35]
Vinblastine	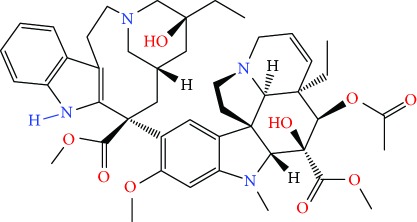	C_46_H_58_N_4_O_9_	810.989	Oral (KB) cancer [35]
*cis*-Annomontacin	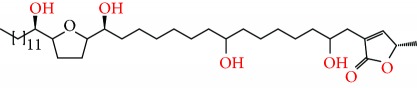	C_37_H_68_O_7_	624.4965	Hepatic (Hep G2 and Hep 2,2,15) cancer [88]
*cis*-Annonacin	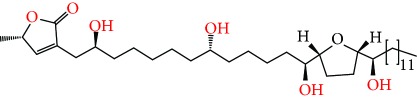	C_35_H_64_O_7_	596.88	Breast (MCF-7), colorectal (HT-29), and lung (A549) cancers [85]
*cis*-Annonacin-10-one	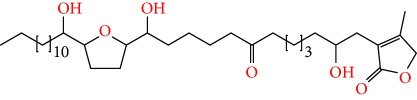	C_35_H_62_O_7_	594.874	Breast (MCF-7), colorectal (HT-29), and lung (A549) cancers [85]
*cis*-Corossolone	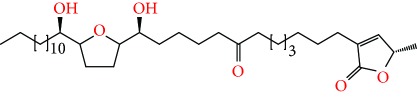	C_35_H_62_O_6_	578.875	Hepatic (Hep G2 and Hep 2,2,15) cancer [81]
*cis*-Goniothalamicin	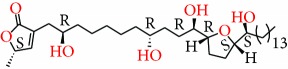	C_35_H_64_O_7_	596.89	Breast (MCF-7), colorectal (HT-29), and lung (A549) cancers [85]

**Table 3:** Anticancer effects of AGEs and extracts derived from the different aerial organs of *A. muricata.*

Cancers	Cell lines	Chemical compound or solvent	Class	Plant part	Dose, IC_50_, ED_50_, GI_50_, LC_50_, IC_25_, and/or MIC	Anticancer effects
Breast cancer	MCF-7	Annomuricin A	AGE	Leaf	ED_50_ > 1.0 *μ*g/mL	Cytotoxic activity [17]
		Annomuricin B				
		Annomuricin C	AGE	Leaf	—–—	Cytotoxic activity [86]
		Annomuricin E	AGE	Leaf	ED_50_ = 1.45 *μ*g/mL	Cytotoxic activity [16]
		Muricatocin C	AGE	Leaf	—–—	Cytotoxic activity [86]
		Muricapentocin	AGE	Leaf	ED_50_ = 1.90 *μ*g/mL	Cytotoxic activity [16]
		Annomutacin	AGE	Leaf	ED_50_ > 1.0 *μ*g/mL	Cytotoxic activity [43]
		(2,4-*cis*)-10R-Annonacin-A-one + (2,4-*trans*)-10R-annonacin-A-one	AGE	Leaf	ED_50_ = 5.70 × 10^−1^ *μ*g/mL	
		Annohexocin	AGE	Leaf	ED_50_ = 2.26 *μ*g/mL	Significant cytotoxic activity [34]
		Muricatocin A	AGE	Leaf	ED_50_ = 1.23 × 10^−1^ *μ*g/mL	Cytotoxic activity [44]
		Muricatocin B	AGE	Leaf	ED_50_ = 1.03 × 10^−1^ *μ*g/mL	
		Annopentocin A	AGE	Leaf	ED_50_ = 17.93 *μ*g/mL	Cytotoxic activity [32]
		Annopentocin B	AGE	Leaf	ED_50_ = 3.56 *μ*g/mL	
		Annopentocin C	AGE	Leaf	ED_50_ = 2.97 *μ*g/mL	
		*cis*-Annomuricin-D-one + *trans*-annomuricin-D-one	AGEs	Leaf	ED_50_ = 6.11 × 10^−1^ *μ*g/mL	
		Murihexocin A	AGE	Leaf	ED_50_ = 12.54 *μ*g/mL	Significant cytotoxic activity [15]
		Murihexocin B	AGE	Leaf	ED_50_ = 6.95 *μ*g/mL	
		Murihexocin C	AGE	Seed	ED_50_ = 3.8 *μ*g/mL	Cytotoxic activity [47]
		Muricoreacin	AGE	Seed	ED_50_ = 1.3 *μ*g/mL	
		Muricatacin	AGE	Seed	ED_50_ = 9.8 *μ*g/mL	Cytotoxic activity [48]
		Isoannonacin	AGE	Seed	IC_50_ = 1.1 × 10^−2^ *μ*g/mL	Cytotoxic activity [49]
		Isoannonacin-10-one	AGE	Seed	IC_50_ = 1.4 × 10^−2^ *μ*g/mL	
		Goniothalamicin	AGE	Seed	IC_50_ = 5.7 × 10^−2^ *μ*g/mL	
		Gigantetrocin	AGE	Seed and/or leaf	IC_50_ = 2.3 × 10^−2^ *μ*g/mL	
		Gigantetrocin A	AGE	Seed	ED_50_ = 5.3 × 10^−1^ *μ*g/mL	Cytotoxic activity [50]
		Muricatetrocin A	AGE	Seed and/or leaf	ED_50_ = 1.03 *μ*g/mL	Cytotoxic activity [17, 50]
		Muricatetrocin B	AGE	Seed	ED_50_ = 1.86 *μ*g/mL	
		Gigantetrocin B	AGE	Seed	ED_50_ = 5.3 × 10^−1^ *μ*g/mL	
		*cis*-Annonacin	AGE	Seed	IC_50_ = 1.18 *μ*g/mL	Cytotoxic activity [85]
		*cis*-Annonacin-10-one	AGE	Seed	IC_50_ = 2.9 × 10^−1^ *μ*g/mL	
		*cis*-Goniothalamicin	AGE	Seed	IC_50_ = 1.05 *μ*g/mL	
		Arianacin	AGE	Leaf	IC_50_ = 4.0 × 10^−1^ *μ*g/mL	
		Javoricin	AGE	Leaf	IC_50_ = 2.3 × 10^−1^ *μ*g/mL	
		Hexane	Extract	Leaf	Doses: 1.56, 3.12, 6.25, 12.5, 25, 50, and 100 *μ*g/mL; IC_50_ = 49.92 ± 2.23 *μ*g/mL	Significantly reduced cell proliferation in cancer cells [31]
		Ethyl acetate	Extract	Fruit	Doses: 1.56, 3.12, 6.25, 12.5, 25, 50, and 100 *μ*g/mL; IC_50_ = 6.39 ± 0.43 *μ*g/mL	
		Methanol	Extract	Leaf	Doses: 1.56, 3.12, 6.25, 12.5, 25, 50, and 100 *μ*g/mL; IC_50_ = 85.58 ± 3.55 *μ*g/mL	
		———	Extract	Leaf	0, 50, 100, 150, and 200 *μ*g/mL; IC_50_ > 200 *μ*g/mL	Inhibited growth of cancer cells [23]
		Ethanol (95%)	Extract	Leaf	GI_50_ = 6.2 *μ*g/mL	Cytotoxic activity [45]
		(+)-(3S,6S,7R,8S)-Periconone A	Fungal strain Extract	Leaf	0.01–10 *μ*mol/mL	Cytotoxic activity [38]
		(−)-(1R,4R,6S,7S)-2-Caren-4,8-olide				
	MDA-MB-231	Hexane	Extract	Leaf	Doses: 1.56, 3.12, 6.25, 12.5, 25, 50, and 100 *μ*g/mL; IC_50_ = 38.72 ± 0.99 *μ*g/mL	Significantly reduced cell proliferation in cancer cells [31]
		Ethyl acetate	Extract	Fruit	Doses: 1.56, 3.12, 6.25, 12.5, 25, 50, and 100 *μ*g/mL; IC_50_ = 11.36 ± 0.67 *μ*g/mL	Significantly reduced cell proliferation in cancer cells [31]
		Methanol	Extract	Leaf	Doses: 1.56, 3.12, 6.25, 12.5, 25, 50, and 100 *μ*g/mL; IC_50_ > 100 *μ*g/mL	
		———	Extract	Seed	Doses: 50, 100, 150, and 200 *μ*g/mL; IC_50_ > 200 *μ*g/mL	Inhibited the growth of cancer cells [23]
	MDA-MB-231-pcDNA3	Methanol	Extract	Pericarp	IC_50_ > 80 *μ*g/mL	Cytotoxic activity [24]
		Methanol	Extract	Leaf		
		Methanol	Extract	seed		
	MDA-MB-231-*BCRP* clone 23	Methanol	Extract	Pericarp		
		Methanol	Extract	Seed		
		Methanol	Extract	Leaf		
	———	Ethanolic component (7,12-dimethylbenzeneanthracene (DMBA))	Extract	Fruit	Three groups of albino mice treated intragastrically by gavage for 6 weeks: 20 mg/mL/week of DMBA + 200 mg/mL/day of extract, 20 mg/mL/week of DMBA + 100 mg/mL/day of extract and 20 mg/mL/week of DMBA + 50 mg/mL/day of extract [139]	Prevented DMBA-induced DNA damage [77, 139]
		Leaves boiled in water	Beverage	Leaf	A 66-year-old female who has been diagnosed with cancer used to boil 10–12 dry leaves in water for 5–7 minutes, 8 oz PO daily at that time	Her metastatic breast cancer is still stable after 5 years on graviola and Xeloda after previously progressing on multiple lines of therapy [118]
	MDA-MB-468	———	Extract	Leaf	Doses: 5, 25, 50, or 100 *μ*g/mL; IC_50_ = 4.8 *μ*g/mL*in vitro*. In addition 200 mg/kg/35 week injected into the back of athymic mice *in vivo*	Inhibited EGFR-overexpression and EGFR mRNA expression. Induced cell cycle arrest at the G0/G1 phase. Induced apoptosis through caspase-3 activation*. In vivo*, it inhibited the growth of MDA-MB-468 tumors implanted in athymic mice (32% growth inhibition). It also significantly reduced the protein expression of EGFR, p-ERK, and p-EGFR in tumors [23]
	MDA	Ethanol	Extract	Leaf	IC_50_ = 248.77 *μ*g/mL	Cytotoxic activity [93]
	SKBR3	Ethanol	Extract	Leaf	IC_50_ = 202.33 *μ*g/mL	
	T47D	Ethanol	Extract	Fruit	IC_50_ = 17.15 *μ*g/mL	Induced cytotoxicity and apoptosis [22]
Bladder cancer	ECV-304	Ethanol	Extract	Twing	0.1–10 mg/mL *in vitro*, MIC = 2 mg/mL and 0.5 g/kg into albino mice *in vivo* [96]	Cytotoxic activity against cancer cells *in vitro* and within reduction of time reaction *in vivo* [96]
Prostate cancer	PC-3	Muricin J, K, or L	AGEs	Leaf	Dose: 20 *μ*g/mL (24 h)	Antiproliferative activity against human cancer cells [29]
		Annomuricin E	AGE	Leaf	ED_50_ = 1.46 × 10^−1^ *μ*g/mL	Cytotoxic activity [16]
		Muricapentocin	AGE	Leaf	ED_50_ = 4.50 × 10^−1^ *μ*g/mL	
		Annohexocin	AGE	Leaf	ED_50_ = 0.0195 *μ*g/mL	Significant cytotoxic activity [34]
		Annopentocin A	AGE	Leaf	ED_50_ = 1.14 *μ*g/mL	Cytotoxic activity [32]
		Annopentocin B	AGE	Leaf	ED_50_ = 2.12 × 10^−1^ *μ*g/mL	
		Annopentocin C	AGE	Leaf	ED_50_ = 2.28 × 10^−1^ *μ*g/mL	
		*cis*-Annomuricin-D-one + *trans*-annomuricin-D-one	AGEs	Leaf	ED_50_ = 1.32 *μ*g/mL	
		Murihexocin A	AGE	Leaf	ED_50_ = 1.71 × 10^−2^ *μ*g/mL	Significant cytotoxic activity [15]
		Murihexocin B	AGE	Leaf	ED_50_ = 0.126 *μ*g/mL	
		Murihexocin C	AGE	Fruit	ED_50_ = 0.86 *μ*g/mL	Cytotoxic activity [47]
		Muricoreacin			ED_50_ = 0.025 *μ*g/mL	
		Muricin M	AGE	fruit	Dose: 20 *μ*g/mL	Antiproliferative activities against human prostate cancer cells [30]
		Muricin N	AGEs	Leaf		
		Muricenin				
	———	Water	Extract	Leaf	F344 male rats (≈200 g) were gavaged 30 mg/mL (10 rats) and 300 mg/mL (10 rats) and fed ad libitum alongside 10 control rats for two months	Reduced prostate size *in vivo*, possibly through apoptosis [21]
Colorectal cancer	HT-29	Annomuricin A	AGE	Leaf	ED_50_ > 1.0 *μ*g/mL	Cytotoxic activity [17]
		Annomuricin B	AGE	Leaf	ED_50_ = 4.35 × 10^−1^ *μ*g/mL	
		Annomuricin C	AGE	Leaf	—–—	Cytotoxic activity [86]
		Muricatocin C				
		Annomuricin E	AGE	Leaf	Doses: 1, 2, 4, 8, and 16 *μ*g/mL [25]; ED_50_ = 6.68 × 10^−2^ *μ*g/mL [16]; IC_50_: 5.72 ± 0.41 *μ*g/mL (12 hr), 3.49 ± 0.22 *μ*g/mL (24 hr), and 1.62 ± 0.24 *μ*g/mL (48 hr) [25].	Induced toxicity against cancer cells [16, 25]. Suppressed proliferation of cancer cells and induced lactate dehydrogenase leakage, cell cycle arrest at G1 phase, and apoptosis mediated through activation of caspases 3/7 and 9. Also induced a time-dependent upregulation of Bax and downregulation of Bcl-2 at both the mRNA and protein level [25]
		Muricapentocin	AGE	Leaf	ED_50_ = 7.10 × 10^−2^ *μ*g/mL	Cytotoxic activity [16]
		Annomutacin	AGE	Leaf	ED_50_ > 1.0 *μ*g/mL	Cytotoxic activity [43]
		(2,4-*cis*)-10R-Annonacin-A-one + (2,4-*trans*)-10R-annonacin-A-one	AGE	Leaf	ED_50_ > 1.0 *μ*g/mL	
		Annohexocin	AGE	Leaf	ED_50_ = 0.78 *μ*g/mL	Significant cytotoxic activity [34]
		Muricatocin A	AGE	Leaf	ED_50_ = 1.56 *μ*g/mL	Cytotoxic activity [44]
		Muricatocin B	AGE	Leaf	ED_50_ = 1.66 *μ*g/mL	
		Annopentocin A	AGE	Leaf	ED_50_ = 1.63 *μ*g/mL	Cytotoxic activity [32]
		Annopentocin B	AGE	Leaf	ED_50_ = 1.64 *μ*g/mL	
		Annopentocin C	AGE	Leaf	ED_50_ = 1.24 *μ*g/mL	
		*cis*-Annomuricin-D-one + *trans*-annomuricin-D-one	AGEs	Leaf	ED_50_ < 10^−2^ *μ*g/mL	
		Murihexocin A	AGE	Leaf	ED_50_ = 3.00 *μ*g/mL	Significant cytotoxic activity [15]
		Murihexocin B	AGE	Leaf	ED_50_ = 2.30 *μ*g/mL	
		Murihexocin C	AGE	Seed	ED_50_ = 1.3 *μ*g/mL	Cytotoxic activity [47]
		Muricoreacin	AGE	Seed	ED_50_ = 0.57 *μ*g/mL	
		Muricatacin	AGE	Seed	ED_50_ = 14.0 *μ*g/mL	Cytotoxic activity [48]
		Isoannonacin	AGE	Seed	IC_50_ < 10^−3^ *μ*g/mL	Cytotoxic activity [49]
		Isoannonacin-10-one	AGE	Seed	IC_50_ = 1.8 × 10^3^ *μ*g/mL	
		Goniothalamicin	AGE	Seed	IC_50_ = 1.1 × 10^−3^ *μ*g/mL	
		Gigantetrocin	AGE	Seed and/or leaf	IC_50_ < 10^3^ *μ*g/mL	
		Gigantetrocin A	AGE	Seed	ED_50_ < 10^−8^ *μ*g/mL	Cytotoxic activity [50]
		Muricatetrocin A	AGE	Seed and/or leaf	ED_50_ < 10^−8^ *μ*g/mL	Cytotoxic activity [17, 50]
		Muricatetrocin B	AGE	Seed	ED_50_ = 2.8 × 10^−5^ *μ*g/mL	
		Gigantetrocin B	AGE	Seed	ED_50_ = 4.1 × 10^−5^ *μ*g/mL	
		*cis*-Annonacin	AGE	Seed	IC_50_ = 1.0 × 10^−8^ *μ*g/mL	Cytotoxic activity [85]
		*cis*-Annonacin-10-one	AGE	Seed	IC_50_ = 9.0 × 10^−4^ *μ*g/mL	
		*cis*-Goniothalamicin	AGE	Seed	IC_50_ = 5.3 × 10^−3^ *μ*g/mL	
		Arianacin	AGE	Leaf	IC_50_ = 4.4 *μ*g/mL	
		Javoricin	AGE	Leaf	IC_50_ = 1.8 *μ*g/mL	
		Hexane	Extract	Leaf	Doses: 10, 20, 40, and 80 *μ*g/mL, IC_50_ = 14.93 ± 0.6 *μ*g/mL (72 hr)	Significantly reduced cell proliferation in cancer cells [26]
		Ethyl acetate	Extract	Leaf	Doses *in vitro*: 10, 20, 40, and 80 *μ*g/mL [26]; 0.62, 1.25, 2.5, 5, 10, 20, 40, and 80 *μ*g/mL [25]; IC_50_ = 4.29 ± 0.24 *μ*g/mL (72 hr) [26]. *Doses in vivo:* 250 or 500 mg/kg into male Sprague-Dawley rats [25].	Induced significant cytotoxic effects, cell cycle arrest at G1 phase, and apoptosis. Treatment also caused excessive accumulation of ROS followed by disruption of MMP, cytochrome c leakage, and activation of the initiator and executioner caspases in cancer cells. In addition, it upregulated Bax and downregulated Bcl-2 proteins. Furthermore, treatment conspicuously blocked the migration and invasion of cancer cells [26]. In rats treated with azoxymethane to induce colorectal carcinogenesis. This extract reduced colonic aberrant crypt foci formation by 72.5% *in vivo* via downregulation of PCNA and Bcl-2 proteins and upregulation of Bax protein as well as an increase in the levels of enzymatic antioxidants and a decrease in the malondialdehyde level of the colon tissue homogenates, suggesting the suppression of lipid peroxidation [25]
		Methanol	Extract	Leaf	Doses: 10, 20, 40, and 80 *μ*g/mL and IC_50_ > 100 *μ*g/mL (72 hr)	Significantly reduced the cell proliferation in cancer cells [26]
	HCT-116	Hexane	Extract	Leaf	Doses: 10, 20, 40, and 80 *μ*g/mL and IC_50_ = 12.26 ± 0.42 *μ*g/mL (72 hr)	
		Ethyl acetate	Extract	Leaf	Doses: 10, 20, 40, and 80 *μ*g/mL and IC_50_ = 3.91 ± 0.35 *μ*g/mL (72 hr)	In cancer cells, induced significant cytotoxic effects, cell cycle arrest at the G1 phase, and apoptosis as well as excessive accumulation of ROS followed by disruption of MMP, cytochrome c leakage, and activation of the initiator and executioner caspases. It also upregulated Bax and downregulated Bcl-2 protein. Furthermore, treatment conspicuously blocked the migration and invasion of cancer cells [26]
		Methanol	Extract	Seed	Doses: 10, 20, 40, and 80 *μ*g/mL and IC_50_ > 100 *μ*g/mL (72 hr)	Significantly reduced cell proliferation in cancer cells [26]
	HCT116 (*p53*^+*/*+^)	Methanol	Extract	Pericarp	IC_50_ > 100 *μ*g/mL	Cytotoxic activity [24]
		Methanol	Extract	Leaf	IC_50_ > 80 *μ*g/mL	
		Methanol	Extract	Seed		
	HCT116 (*p53*^−*/*−^)	Methanol	Extract	Pericarp		
		Methanol	Extract	Leaf		
		Methanol	Extract	Leaf		
	———	Ethanolic	Extract	Leaf	300 mg/kg into Wistar albino rats	Showed potent anticancer activity through apoptosis and reduction of aberrant crypt foci formation [20]
		Ethanol	Extract	Leaf	100 mg/kg body weight/4 weeks are administrated into Wistar rats	In a rat model of *Cycas*-induced colorectal carcinogenesis, protected against some early events as monitored by histology and protein expression [140]
	COLO-205	96% Ethanol [112] or ethanol soluble fraction leaf water extract contains 0.36% acetogenin (*w*/*w*) or 3.6 mg/g, and a 10 g water extract is equivalent to a 2 g ethanolic fraction [94].	Extract	Leaf	Doses *in vitro*: 400, 200, 100, 50, 25, 12.5, 6.25, 3.125, and 1.5625 mg/L, IC_50_ = 189.6 *μ*g/mL (48 hr) [112]. Ex vivo, the colorectal cancer patients consumed either 300 mg of the extract, or maltose as a placebo, in the form of a capsule after breakfast [94].	Enhanced proapoptotic caspase-3 marker activity [112]. Ex vivo and clinical studies showed higher cytotoxicity in the supplemented group compared with the placebo group [94]
	DLD-1	Ethanol soluble fraction leaf water extract contains 0.36% acetogenin (*w*/*w*) or 3.6 mg/g, and a 10 g water extract is equivalent to a 2 g ethanolic fraction.	Extract	Leaf	Patients consumed either 300 mg of extract, or maltose as a placebo, in the form of a capsule after breakfast.	Ex vivo and clinical studies showed higher cytotoxicity in the supplemented group compared with the placebo group [94]
	HTC-8	(+)-(3S,6S,7R,8S)-Periconone A	Fungal strain Extract	Leaf	Doses: 0.01–10 *μ*mol/ml	Cytotoxic activity [38]
		(−)-(1R,4R,6S,7S)-2-Caren-4,8-olide	Fungal strain Extract	Leaf		
Lung cancer	A549	Annomuricin A	AGE	Leaf	ED_50_ = 3.30 × 10^−1^ *μ*g/mL	Cytotoxic activity [17]
		Annomuricin B	AGE	Leaf	ED_50_ = 1.59 × 10^−1^ *μ*g/mL	
		Annomuricin C	AGE	Leaf	—–—	Cytotoxic activity [86]
		Muricatocin C	AGE	Leaf		
		Annomuricin E	AGE	Leaf	ED_50_ = 1.12 × 10^−1^ *μ*g/mL	Cytotoxic activity [16]
		Muricapentocin	AGE	Leaf	ED_50_ = 1.93 × 10^−1^ *μ*g/mL	
		Annomutacin	AGE	Leaf	ED_50_ = 1.57 × 10^−1^ *μ*g/mL	Cytotoxic activity [43]
		(2,4-*cis*)-10R-Annonacin-A-one + (2,4-*trans*)-10R-annonacin-A-one	AGEs	Leaf	ED_50_ = 1.74 × 10^−1^ *μ*g/mL	
		Annohexocin	AGEs	Leaf	ED_50_ = 0.34 *μ*g/mL	Significant cytotoxic activity [34]
		Muricatocin A	AGE	Leaf	ED_50_ = 7.55 × 10^−2^ *μ*g/mL	Cytotoxic activity [44]
		Muricatocin B	AGE	Leaf	ED_50_ = 3.34 × 10^−1^ *μ*g/mL	
		Annopentocin A	AGE	Leaf	ED_50_ = 1.71 × 10^−1^ *μ*g/mL	Cytotoxic activity [32]
		Annopentocin B	AGE	Leaf	ED_50_ = 2.74 × 10^−2^ *μ*g/mL	
		Annopentocin C	AGE	Leaf	ED_50_ = 2.06 × 10^−2^ *μ*g/mL	
		*cis*-Annomuricin-D-one + *trans*-annomuricin-D-one	AGEs	Leaf	ED_50_ < 10^−2^ *μ*g/mL	
		Murihexocin A	AGE	Leaf	ED_50_ = 1.32 *μ*g/mL	Significant cytotoxic activity [15]
		Murihexocin B	AGE	Leaf	ED_50_ = 1.08 *μ*g/mL	
		Murihexocin C	AGE	Seed	ED_50_ = 1.1 *μ*g/mL	Cytotoxic activity [47]
		Muricoreacin	AGE	Seed	ED_50_ = 0.23 *μ*g/mL	
		Muricatacin	AGE	Seed	ED_50_ = 23.3 *μ*g/mL	Cytotoxic activity [48]
		Isoannonacin	AGE	Seed	IC_50_ = 9.6 × 10^−3^ *μ*g/mL	Cytotoxic activity [49]
		Isoannonacin-10-one	AGE	Seed	IC_50_ = 9.7 × 10^3^ *μ*g/mL	
		Goniothalamicin	AGE	Seed and/or leaf	IC_50_ = 8.0 × 10^−3^ *μ*g/mL	
		Gigantetrocin	AGE	Seed and/or leaf	IC_50_ < 10^−3^ *μ*g/mL	
		Gigantetrocin A	AGE	Seed	ED_50_ = 8.1 × 10^−3^ *μ*g/mL	Cytotoxic activity [17, 50]
		Muricatetrocin A	AGE	Seed and/or leaf	ED_50_ = 1.4 × 10^−1^ *μ*g/mL	
		Muricatetrocin B	AGE	Seed	ED_50_ = 4.9 × 10^−1^ *μ*g/mL	
		Gigantetrocin B	AGE	Seed	ED_50_ = 2.5 × 10^−1^ *μ*g/mL	
		*cis*-Annonacin	AGE	Seed	IC_50_ = 2.3 × 10^−1^ *μ*g/mL	Cytotoxic activity [85]
		*cis*-Annonacin-10-one	AGE	Seed	IC_50_ = 3.5 × 10^−1^ *μ*g/mL	
		*cis*-Goniothalamicin	AGE	Seed	IC_50_ = 1.3 × 10^−1^ *μ*g/mL	
		Arianacin	AGE	Leaf	IC_50_ = 4.7 × 10^−3^ *μ*g/mL	
		Javoricin	AGE	Leaf	IC_50_ = 1.7 × 10^−2^ *μ*g/mL	
		Ethyl acetate component	Extract	Leaf	Doses: 1.56, 3.12, 6.25, 12.5, 25, 50, and 100 *μ*g/mL; IC_50_: 5.09 ± 0.41 *μ*g/mL (72 hr)	Selective cytotoxic effect against cancer cells and significant lactate dehydrogenase leakage and phosphatidylserine externalization demonstrated by fluorescence analysis. Treatment also elevated ROS formation, while attenuating MMP via upregulation of Bax and downregulation of Bcl-2. This was accompanied by cytochrome c release to the cytosol, which triggered activation of caspase-9 and caspase-3. These proapoptotic effects were accompanied by cell cycle arrest at the G0/G1 phase and suppression of NF-*κ*B translocation from the cytoplasm to the nucleus [31]
		Hexane	Extract	Leaf	Doses: 1.56, 3.12, 6.25, 12.5, 25, 50, and 100 *μ*g/mL; IC_50_ = 21.05 ± 0.42 *μ*g/mL	Significantly reduced cell proliferation in cancer cells [31]
		Methanol	Extract	Leaf	Doses: 1.56, 3.12, 6.25, 12.5, 25, 50, and 100 *μ*g/mL; IC_50_ > 100 *μ*g/mL	
		(+)-(3S,6S,7R,8S)-Periconone A	Fungal strain Extract	Leaf	Doses: 0.01–10 *μ*mol/mL	Cytotoxic activity [38]
		(−)-(1R,4R,6S,7S)-2-Caren-4,8-olide	Fungal strain Extract	Leaf		
	H-460	Ethanol	Extract	Tree/Leaf	IC_50_ < 0.22 *μ*g/mL	Cytotoxic activity [95]
		Ethanol (95%)	Extract	Pericarp	GI_50_ = 4.0 *μ*g/mL	Cytotoxic activity [45]
	NCI-H292	Methanol	Extract	Pericarp	IC_50_: 24.94 ± 0.74 *μ*g/mL	Antiproliferative and cytotoxic activities towards cancer cells [113]
Leukemia (hematological malignancies)	U-937	Annonacin A	AGE	Pericarp	Doses: 0.1, 0.46, and 1.0 mg/mL	Cytotoxic activity [46]
		Annomuricin A	AGE	Pericarp		
		Methanol	Extract	Pericarp	MEC > 1 mg/mL	
		Hexane	Extract	Leaf	MEC = 1 mg/mL	
		Ethyle acetate	Extract	Stem	MEC = 0.1 mg/mL	
		Ethyle acetate	Extract	Stem	LC_50_ = 7.8 ± 0.3 *μ*g/mL	Cytotoxic activity [131]
		Ethyle acetate	Extract	Stem	IC_50_ = 10.5 ± 2.3 and/or 28.1 ± 13.0 *μ*g/mL	Cytotoxic activity [33]
		Methanol	Extract	Stem	IC_50_ = 60.9 ± 10.4 and/or 38.5 ± 8.6 *μ*g/mL	
		Hexane	Extract	Stem	IC_50_ = 18.2 ± 0.8 and/or 15.7 ± 5.1 *μ*g/mL	
	K562	Ethanol	Extract	Leaf	Doses *in vitro*: 0.625 mg/mL, 1.25 mg/mL, 2.5 mg/mL, and 5.0 mg/mL [19]; 0.1–10 mg/mL [96]; MIC = 7 mg/mL [96]. Dose *in vivo*: 0.5 g/kg into albino mice [96].	Showed cytotoxicity *in vitro* [19] and *in vivo* [96]. This was accompanied *in vitro* by significantly increased caspase-3 activity. Induction of apoptosis was confirmed by a terminal deoxynucleotidyl transferase-mediated dUTP nick-end labelling (TUNEL) assay [19]
	HL-60	Ethanol	Extract	Root	IC_50_ = 14 ± 2.4 *μ*g/mL	Induced apoptosis through loss of MMP and inhibited proliferation via G0/G1 cell cycle arrest [27]
		Ethanol	Extract	Fruit/pericarp	IC_50_ = 49 ± 3.2 *μ*g/mL	
		Ethanol	Extract	Leaf	IC_50_ = 9 ± 0.8 *μ*g/mL	
	CCRF-CEM	Methanol	Extract	Seed	IC_50_ = 4.58 ± 0.25 *μ*g/mL	Induced cytotoxic, apoptosis, and cell cycle arrest [24]
		Methanol	Extract	Leaf	IC_50_ = 0.57 ± 0.02 *μ*g/mL	
		Methanol	Extract	Seed	IC_50_ = 0.36 ± 0.03 *μ*g/mL	
	CEM/ADR5000	Methanol	Extract	Pericarp	IC_50_ = 5.25 ± 0.38 *μ*g/mL	
		Methanol	Extract	Leaf	IC_50_ = 6.65 ± 0.22 *μ*g/mL	
		Methanol	Extract	Leaf	IC_50_ = 23.70 ± 1.64 *μ*g/mL	
Renal cancer	A498	Annomuricin E	AGE	Leaf	ED_50_ = 1.41 *μ*g/mL	Cytotoxic activity [16]
		Muricapentocin	AGE	Leaf	ED_50_ = 1.72 *μ*g/mL	
		Annohexocin	AGE	Leaf	ED_50_ = 2.36 *μ*g/mL	Cytotoxic activity [34]
		Annopentocin A	AGE	Leaf	ED_50_ = 6.07 × 10^−1^ *μ*g/mL	Cytotoxic activity [32]
		Annopentocin B	AGE	Leaf	ED_50_ = 3.79 × 10^−1^ *μ*g/mL	
		Annopentocin C	AGE	Leaf	ED_50_ = 2.58 × 10^−1^ *μ*g/mL	
		*cis*-Annomuricin-D-one + *trans*-annomuricin-D-one	AGEs	Leaf	ED_50_ = 1.22 × 10^−1^ *μ*g/mL	
		Murihexocin A	AGE	Leaf	ED_50_ = 2.51 *μ*g/mL	Significant cytotoxic activity [15]
		Murihexocin B	AGE	Leaf	ED_50_ = 4.92 *μ*g/mL	
		Murihexocin C	AGE	Leaf	ED_50_ = 2.5 *μ*g/mL	Cytotoxic activity [47]
		Muricoreacin	AGE	Leaf	ED_50_ = 0.71 *μ*g/mL	
Pancreatic cancer	PACA	Annomuricin E	AGE	Leaf	ED_50_ = 2.42 × 10^−2^ *μ*g/mL	Cytotoxic activity [16]
		Muricapentocin	AGE	Leaf	ED_50_ = 5.03 × 10^−2^ *μ*g/mL	
	PACA-2	Annohexocin	AGE	Leaf	ED_50_ = 0.77 *μ*g/mL	Significant cytotoxic activity [34]
		Annopentocin A	AGE	Leaf	ED_50_ = 3.58 × 10^−2^ *μ*g/mL	Cytotoxic activity [32]
		Annopentocin B	AGE	Leaf	ED_50_ = 1.62 × 10^−1^ *μ*g/mL	
		Annopentocin C	AGE	Leaf	ED_50_ = 4.28 × 10^−1^ *μ*g/mL	
		*cis*-Annomuricin-D-one + *trans*-annomuricin-D-one	AGEs	Leaf	ED_50_ < 10^−2^ *μ*g/mL	
		Murihexocin A	AGE	Leaf	ED_50_ = 9.73 × 10^−2^ *μ*g/mL	Significant cytotoxic activity [15]
		Murihexocin B	AGE	Leaf	ED_50_ = 0.413 *μ*g/mL	
		Murihexocin C	AGE	Leaf and/or stem	ED_50_ = 0.49 *μ*g/mL	Cytotoxic activity [47]
		Muricoreacin	AGE	Leaf and/or stem	ED_50_ = 2.3 *μ*g/mL	
	FG/COLO357	Powder without binders or fillers (capsule contents is suspended in DMSO (100 mg/mL DMSO)	Extract	Leaf	Doses: 10–200 *μ*g/mL. IC_50_ = 200 *μ*g/mL	Induced cytotoxicity and necrosis by inhibiting cellular metabolism. In addition, it downregulated the expression of molecules related to hypoxia and glycolysis (i.e., HIF-1*α*, NF-*κ*B, GLUT1, GLUT4, HKII, and LDHA) in cancer cells. Also, the motility of pancreatic cancer cells was decreased [28]
	CD18/HPAF	DMSO *in vitro* and H_2_O *in vivo*	Extract	Leaf	Doses: 10–200 *μ*g/mL, IC_50_ = 73 *μ*g/mL *in vitro*. 50 mg/kg/35 days injected orthotopically in the pancreas of athymic nude mice	Induced cytotoxicity and necrosis and inhibited cellular metabolism. In addition, it downregulates the expression of molecules related to hypoxia and glycolysis (i.e., HIF-1*α*, NF-*κ*B, GLUT1, GLUT4, HKII, and LDHA) in cancer cells. After treatment, the motility of pancreatic cancer cells was decreased. It also caused 59.8% growth inhibition of pancreatic tumor induced in mice orthotopically implanted with CD18/HPAF cells [28]
	Capan-1	Hexane	Extract	Seed	IC_25_~7.8–8 *μ*g/mL	Inhibited cell proliferation and induced mild cytotoxicity in cancer cells [92]
		DMSO	Commercialized Extract	Seed	IC_25_~0.9–1.0 *μ*g/mL	
Hepatic cancer	Hep G2	Muricin H	AGE	Seed	IC_50_ = 9.51 × 10^−2^ *μ*g/mL	Exhibited significant activity in *in vitro* and cytotoxic assays against human hepatoma cell line [81]
		Muricin I	AGE	Leaf	IC_50_ = 5.09 × 10^−2^ *μ*g/mL	
		*cis*-Annomontacin	AGE	Leaf	IC_50_ = 2.98 × 10^−1^ *μ*g/mL	
		*cis*-Corossolone	AGE	Seed	IC_50_ = 1.65 × 10^−1^ *μ*g/mL	
		Annocatalin	AGE	Leaf	IC_50_ = 5.70 *μ*g/mL	
		Annocatacin A	AGE	Leaf	IC_50_ = 12.11 *μ*g/mL	Significant *in vitro* cytotoxic activity [80]
		Annocatacin B	AGE	Seed	IC_50_ = 3.35 × 10^−2^ *μ*g/mL	
		Methanol	Extract	Pericarp	IC_50_ > 80 *μ*g/mL	Cytotoxic activity [24]
		Methanol	Extract	Seed		
		Methanol	Extract	Seed		
		Muricin A	AGE	Seed	IC_50_ = 5.04 *μ*g/mL	Cytotoxic activity [82]
		Muricin B	AGE	Seed	IC_50_ = 1.78 *μ*g/mL	
		Muricin C	AGE	Seed	IC_50_ = 4.99 *μ*g/mL	
		Muricin D	AGE	Seed	IC_50_ = 6.60 × 10^−4^ *μ*g/mL	
		Muricin E	AGE	Seed	———	
		Muricin F	AGE	Seed	IC_50_ = 4.28 × 10^−2^ *μ*g/mL	
		Muricin G	AGE	Seed	———	
		Muricatetrocins A & B	AGE	Seed	IC_50_ = 4.95 × 10^−2^ *μ*g/mL	
		Longifolicin	AGE	Seed	IC_50_ = 4.04 × 10^−4^ *μ*g/mL	
		Corossolin	AGE	Leaf	IC_50_ = 3.53 × 10^−1^ *μ*g/mL	
		Corossolone	AGE	Leaf	IC_50_ = 4.80 × 10^−1^ *μ*g/mL	
		Hexane	Extract	Leaf	Doses: 1.56, 3.12, 6.25, 12.5, 25, 50, and 100 *μ*g/mL; IC_50_ = 77.92 ± 2.23 *μ*g/mL	Significantly reduced cell proliferation in cancer cells [31]
		Ethyl acetate	Extract	Seed	Doses: 1.56, 3.12, 6.25, 12.5, 25, 50, and 100 *μ*g/mL; IC_50_ = 9.3 ± 0.91 *μ*g/mL	
		Methanol	Extract	Seed	Doses: 1.56, 3.12, 6.25, 12.5, 25, 50, and 100 *μ*g/mL; IC_50_ > 100 *μ*g/mL	
	Hep 2,2,15 (a Hep G2 cell line transfected with HBV)	Muricin H	AGE	Seed	IC_50_ = 1.18 × 10^−2^ *μ*g/mL	Exhibited significant activity in *in vitro* and cytotoxic assays in a human hepatoma cell line [81]
		Muricin I	AGE	Leaf	IC_50_ = 2.22 × 10^−1^ *μ*g/mL	
		*cis*-Annomontacin	AGE	Leaf	IC_50_ = 1.62 × 10^−2^ *μ*g/mL	
		*cis*-Corossolone	AGE	Leaf	IC_50_ = 4.76 × 10^−2^ *μ*g/mL	
		Annocatalin	AGE	Leaf	IC_50_ = 3.48 × 10^−3^ *μ*g/mL	
		Annocatacin A	AGE	Seed	IC_50_ = 8.17 × 10^−1^ *μ*g/mL	Significant *in vitro* cytotoxic activity [80]
		Annocatacin B	AGE	Seed	IC_50_ = 2.22 × 10^−1^ *μ*g/mL	
		Muricin A	AGE	Seed	IC_50_ = 5.13 × 10^−3^ *μ*g/mL	Cytotoxic activity [82]
		Muricin B	AGE	Seed	IC_50_ = 4.29 × 10^−3^ *μ*g/mL	
		Muricin C	AGE	Seed	IC_50_ = 3.87 × 10^−3^ *μ*g/mL	
		Muricin D	AGE	Seed	IC_50_ = 4.80 × 10^−2^ *μ*g/mL	
		Muricin E	AGE	Seed	———	
		Muricin F	AGE	Seed	IC_50_ = 3.86 × 10^−3^ *μ*g/mL	
		Muricin G	AGE	Seed	———	
		Muricatetrocins A & B	AGE	Seed	IC_50_ = 4.83 × 10^−3^ *μ*g/mL	
		Longifolicin	AGE	Seed	IC_50_ = 4.90 × 10^−3^ *μ*g/mL	
		Corossolin	AGE	Leaf	IC_50_ = 2.34 × 10^−1^ *μ*g/mL	
		Corossolone	AGE	Leaf	IC_50_ = 2.84 × 10^−1^ *μ*g/mL	
	Bel-7402	(+)-(3S,6S,7R,8S)-Periconone A	fungal strain Extract	Seed and/or Leaf	Doses: 0.01–10 *μ*mol/mL	Cytotoxic activity [38]
		(−)-(1R,4R,6S,7S)-2-Caren-4,8-olide	fungal strain Extract	Seed		
Oral cancer	KB	Corossolone	AGE	Seed	ED_50_ = 0.1 *μ*g/mL	Toxicity against oral cancer cells *in vitro* [35, 83]
		Corossolin	AGE	Seed	ED_50_ = 0.003 *μ*g/mL	
		Solamin	AGE	Seed	ED_50_ = 0.3 *μ*g/mL	Toxicity against oral cancer cells *in vitro* [35]
		Murisolin	AGE	Seed	ED_50_ = 0.1 *μ*g/mL	
		Annonacinone	AGE	Seed	ED_50_ = 0.01 *μ*g/mL	
		Annonacin	AGE	Leaf	ED_50_ = 0.0001 *μ*g/mL	
		Vinblastine	AGE	Leaf	ED_50_ = 0.01 *μ*g/mL	
Stomach cancer	C-678	Ethanol	Extract	Leaf	IC_50_ < 0.22 *μ*g/mL	Cytotoxic activity [95]
Melanoma	A375	H_2_O	Extract	Leaf	IC_50_ > 500 *μ*g/mL (24 and 72 hr)	Cytotoxic activity [36]
		Ethanol	Extract	Leaf	IC_50_ = 20 ± 6 *μ*g/mL (24 hr) and 20 ± 7 *μ*g/mL (72 hr)	
		Pentane	Extract	Leaf	IC_50_ = 140 ± 25 *μ*g/mL (24 hr) and 120 ± 8 *μ*g/mL (72 hr)	
	MDA-MB-435S	*n*-Butanolic	Extract	Leaf	IC_50_ = 29.2 *μ*g/mL. 25, 50, 100, 200, and 400 *μ*g/mL	Significant cytotoxic activity [89, 90]
Skin cancer	———	80% aqueous ethanol	Extract	Leaf	30 mg/kg body weight into ICR mice	Suppressed tumor initiation as well as tumor promotion even at lower dosage [141]
Glioma	SF-268	Ethanol (95%)	Extract	Seed	GI_50_ = 8.5 *μ*g/mL	Cytotoxic activity [45]
	U87MG	Methanol	Extract	Pericarp	IC_50_ > 80 *μ*g/mL	Cytotoxic activity [24]
		Methanol	Extract	Leaf		
		Methanol	Extract	Seed		
	U87MG.Δ*EGFR*	Methanol	Extract	Pericarp		
		Methanol	Extract	Leaf		
		Methanol	Extract	Leaf		
Cervical cancer	HeLa	Ethyl acetate	Extract	Leaf	LC_50_ of (2000 *μ*g/mL) = 131.89%; and LC_50_ of (15.625 *μ*g/mL) = 11.37%	Induced apoptosis [37]
		Ethanol-distillate water	Extract	Leaf	LC_50_ of (2000 *μ*g/mL) = 35.80%; and LC_50_ of (15.625 *μ*g/mL) = 3.97%	
		Chloroform	Extract	Leaf	LC_50_ of (2000 *μ*g/mL) = 65.20%; and LC_50_ of (15.625 *μ*g/mL) = 18.42%	
		*n*-Hexan	Extract	Leaf	LC_50_ of (2000 *μ*g/mL) = 106.53%; and LC_50_ of (15.625 *μ*g/mL) = 21.41%	
	HEp-2 (now HeLa)	Methanol	Extract	Leaf	IC_50_ = 54.92 ± 1.44 *μ*g/mL	Antiproliferative and cytotoxic activities [90, 113]
Ehrlich ascite carcinoma	EACC	Ethanol	Extract	Leaf	IC_50_ = 335.85 *μ*g/mL	Cytotoxic activity *in vitro* [93]
Gastric cancer	BGC-823	(+)-(3S,6S,7R,8S)-Periconone A	Fungal strain Extract	Leaf	Doses: 0.01–10 *μ*mol/mL	Cytotoxic activity [38]
		(−)-(1R,4R,6S,7S)-2-Caren-4,8-olide	Fungal strain Extract	Leaf		
Ovarian cancer	A2780	(+)-(3S,6S,7R,8S)-Periconone A	Fungal strain Extract	Leaf		
		(−)-(1R,4R,6S,7S)-2-Caren-4,8-olide	Fungal strain Extract	Tree/Leaf		
Head and neck squamous cell carcinoma (HNSC)	SCC-25	H_2_O	Extract	Leaf	Doses: 2.5–160 *μ*g/mL; IC_50_ = 12.42 *μ*g/mL	Displayed promising cytotoxic activity and inhibition of cell proliferation via G2M cell cycle arrest [91]

ED50: median effective dose; GI_50_: a concentration for 50% of maximal inhibition of cell proliferation; IC_25_: a concentration causing 50% inhibition; IC_50_: a concentration causing 50% inhibition; LC_50_: a concentration causing 50% cell death; LD: lethal dose; MIC: minimum inhibitory concentration.
